# A *let-7*-to-*miR-125* MicroRNA Switch Regulates Neuronal Integrity and Lifespan in *Drosophila*

**DOI:** 10.1371/journal.pgen.1006247

**Published:** 2016-08-10

**Authors:** Geetanjali Chawla, Padmini Deosthale, Sue Childress, Yen-chi Wu, Nicholas S. Sokol

**Affiliations:** 1 Department of Biology, Indiana University, Bloomington, Bloomington, Indiana, United States of America; 2 Medical Sciences Program, Indiana University, Bloomington, Bloomington, Indiana, United States of America; Princeton, UNITED STATES

## Abstract

Messenger RNAs (mRNAs) often contain binding sites for multiple, different microRNAs (miRNAs). However, the biological significance of this feature is unclear, since such co-targeting miRNAs could function coordinately, independently, or redundantly with one another. Here, we show that two co-transcribed *Drosophila* miRNAs, *let-7* and *miR-125*, non-redundantly regulate a common target, the transcription factor Chronologically Inappropriate Morphogenesis (Chinmo). We first characterize novel adult phenotypes associated with loss of both *let-7* and *miR-125*, which are derived from a common, polycistronic transcript that also encodes a third miRNA, *miR-100*. Consistent with the coordinate upregulation of all three miRNAs in aging flies, these phenotypes include brain degeneration and shortened lifespan. However, transgenic rescue analysis reveal separable roles for these miRNAs: adult *miR-125* but not *let-7* mutant phenotypes are associated with ectopic Chinmo expression in adult brains and are suppressed by *chinmo* reduction. In contrast, *let-7* is predominantly responsible for regulating *chinmo* during nervous system formation. These results indicate that *let-7* and *miR-125* function during two distinct stages, development and adulthood, rather than acting at the same time. These different activities are facilitated by an increased rate of processing of *let-7* during development and a lower rate of decay of the accumulated *miR-125* in the adult nervous system. Thus, this work not only establishes a key role for the highly conserved *miR-125* in aging. It also demonstrates that two co-transcribed miRNAs function independently during distinct stages to regulate a common target, raising the possibility that such biphasic control may be a general feature of clustered miRNAs.

## Author Summary

Deregulation of mRNAs that are targeted by multiple miRNAs is a common feature of a number of diseased states including neurodegenerative disorders. The currently accepted model is that the combined action of all binding miRNAs ensures target repression. Here, we show that two co-expressed miRNAs exert distinct outcomes on a common target. While *miR-125* extends lifespan by repressing its target, *chinmo*, in adult brains, *let-7* downregulates Chinmo in developing animals. Our results indicate that differential processing and turnover rates of *let-7* and *miR-125* contribute to this switch in miRNA activity. This study has identified the physiological relevance of the targeting of a single mRNA by multiple miRNAs in a scenario where each miRNA exerts a distinct and non-overlapping outcome.

## Introduction

RNA-mediated post-transcriptional mechanisms regulate the accumulation and homeostasis of proteins not only during animal development but also during adulthood [1–3]. These mechanisms include regulation by microRNAs (miRNAs), a class of small non-coding RNAs that usually silence messenger RNAs (mRNAs) by binding to partially complementary sequences frequently found in the target 3’ untranslated (3’UTR) sequence [4]. Some miRNAs are known to affect lifespan by post-transcriptionally silencing mRNAs that play critical, beneficial roles at early stages of the life cycle but are deleterious when expressed inappropriately at later stages [1, 2, 5–7]. For example, loss of *C*. *elegans lin-4*, the first miRNA to be functionally characterized for its role in lifespan, leads to shortened lifespan due to the persistence of its target, *lin-14* [2, 8]. Similarly, the adult onset of *Drosophila miR-34* promotes longevity and maintains neuronal homeostasis by repressing *Eip74EF*, a transcription factor required for progression through earlier life stages [1, 3]. Although loss of other miRNAs like *Drosophila miR-1000* lead to shortened lifespan [3], the complete repertoire of miRNAs that regulate aging processes remains uncharacterized [9].

Understanding the role of miRNAs in the adult nervous system is particularly relevant to aging, since the nervous system is a key coordinator of age-related changes in overall organismal physiology [1, 10, 11]. For example, the ablation of specific neurons in both worms and flies extends lifespan [12, 13]. In addition, conserved mechanisms that regulate organismal aging, including insulin signaling and mitochondrial function, modulate the pathology of neurodegenerative disease models [14–18]. Since premature loss of miRNAs has been linked to defective neuronal function and survival as well as the accumulation of disease related proteins, miRNA regulatory networks likely constitute an important component of the normal aging process in the brain [3, 19–21]. Thus, exploring the functional roles of miRNAs and their mRNA targets in the adult brain is necessary to understand the mechanisms involved in the onset and progression of late onset neurodegenerative diseases.

Multiple miRNAs are frequently predicted to regulate the same mRNA indicating that miRNA activity within tissues such as the nervous system is coordinated. Bioinformatic analyses estimate that greater than 70% of targeted human mRNAs and between 30 to 50% of targeted *Drosophila* mRNAs have sites for two or more miRNAs [22–24]. The *Drosophila* predictions are likely underestimates of the frequency of co-targeting in the nervous system, since they were generated prior to the discovery of dozens of *Drosophila* miRNAs as well as the 3’UTR extensions of numerous neural mRNAs [25–27]. Recent analyses have found that co-targeting is particularly prevalent for clustered miRNAs, which are likely to be co-transcribed and therefore co-expressed [28]. Based on reporter assays showing a positive correlation between the number of miRNA sites in a 3’UTR and the degree of its repression [29, 30], the current model suggests that miRNA activity is additive and predicts that spatially overlapping combinations of miRNAs–presumably including those that are co-transcribed–lead to greater target repression [31, 32]. However, there are very few published investigations that have tested this model directly by delineating the individual activities of multiple co-targeting miRNAs. Here, we re-evaluate this model by distinguishing the effects of two co-transcribed neural miRNAs, *let-7* and *miR-125*, on a common target mRNA during development and adulthood.

## Results

### *let-7-Complex* miRNAs modulate age-associated processes in the brain

**Fig 1:**
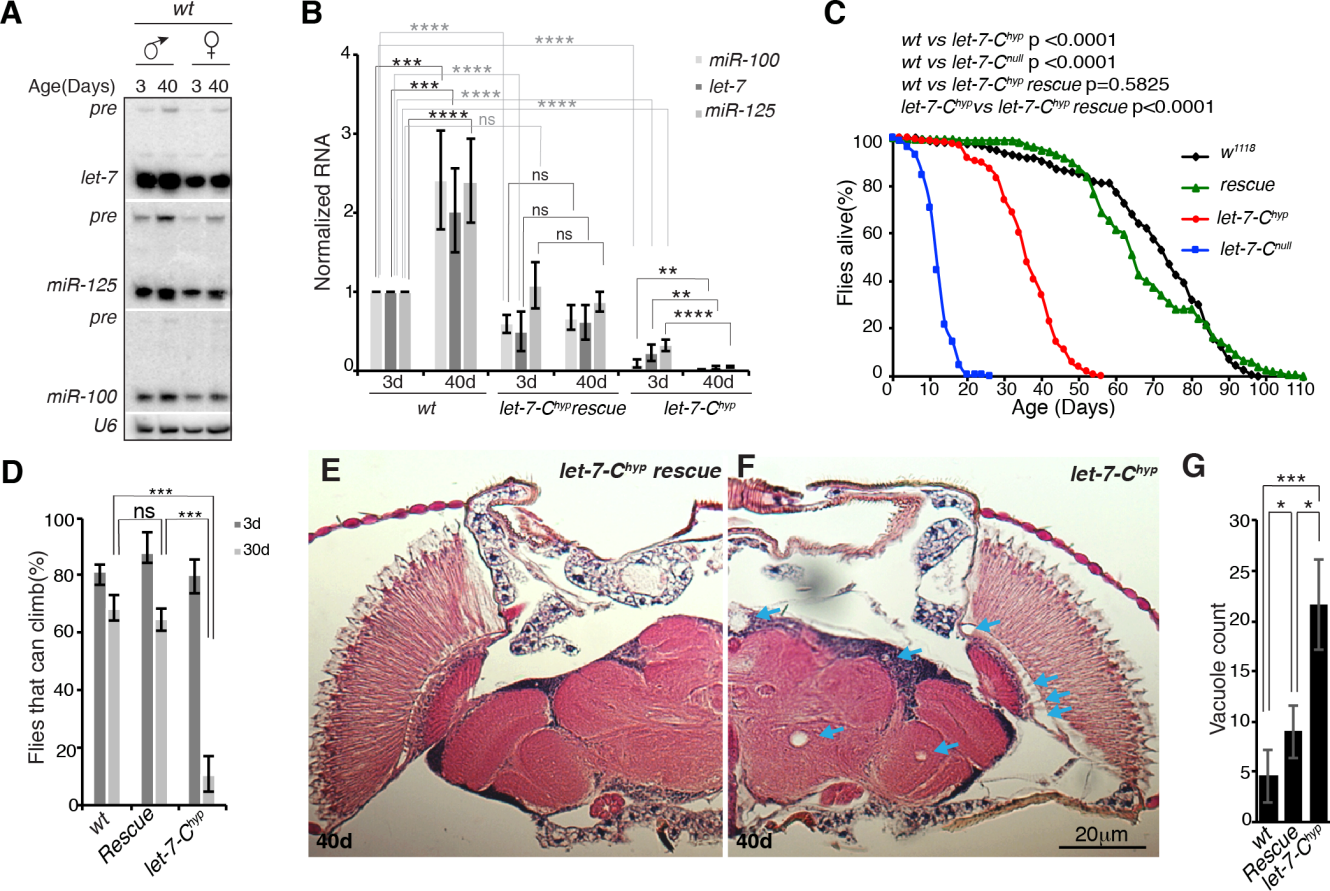
*let-7-C* mutants are short-lived and display adult onset defects in motility and brain morphology. (**A**) Northern blots of total RNA from *w*^*1118*^ (*wt*) males and females that were aged for 3 or 40 days and probed for *let-7*, *miR-125*, and *miR-100*. Blots were also probed with U6 as a loading control. (**B**) Quantitation of *miR-100*, *let-7*, and *miR-125* in 3 or 40 day *w*^*1118*^ (*wt*), *let-7-C*^*hyp*^ rescue, and *let-7-C*^*hyp*^ adult males (n = 3). Processed miRNA levels were normalized to 2S rRNA. (**C**) *let-7-C* mutant males displayed a reduced lifespan (*w*^*1118*^: median survival 74d, maximum lifespan 98d, n = 210 male flies; *let-7-C null*: median survival 12d, maximum lifespan 26d, n = 210 male flies; *let-7-C*^*hyp*^: median survival 36d, maximum lifespan 56d, n = 210 male flies; *let-7-C*^*hyp*^ rescue: median survival 66d, maximum lifespan 110d, n = 210 male flies). P value of the lifespan curves were calculated by log-rank test. (**D**) Climbing activity of 3 or 30 day *w*^*1118*^, *let-7-C*^*hyp*^ rescued and *let-7-C*^*hyp*^ adult males. At 3 days, the climbing ability of *let-7-C*^*hyp*^ (80 ± 5.8%) was comparable to the control *w*^*1118*^ (81.33 ± 2.3%). However, at 30 days, only 10.7 ± 6.18% of *let-7-C*^*hyp*^ males were able to climb as opposed to 68.72.5 ± 4.67% of *w*^*1118*^ males. The age related climbing defects of *let-7-C*^*hyp*^ were almost completely rescued by a single copy of the *let-7-C* rescue transgene (64.66 ± 3.69%). Mean ± S.D of three experiments, n = 15 male flies in each experiment, *** = p value <0.001. (**E**, **F**) Brain sections of 40d *let-7-C*^*hyp*^ rescue (**E**) and *let-7-C*^*hyp*^ mutant males (**F**) (arrows point at vacuoles). (**G**) Quantification of vacuole number. See S1 Table for detailed genotypes. Data represent mean ± S.D (**B**, **D**, **G**).

The *let-7-Complex* (*let-7-C*) locus in *Drosophila* encodes an evolutionarily conserved cluster of three co-transcribed miRNAs: *miR-100*, *let-7* and *miR-125*, the orthologue of *C*. *elegans lin-4* [33, 34]. Although the levels of processed *let-7* are known to increase with age in testes and ovaries [35, 36], the relative expression levels of all three miRNAs have not been characterized in aging flies. To address this, we performed Northern blot and quantitative reverse transcription polymerase chain reaction (qRT-PCR) analyses of whole animals (Fig 1A and 1B, left). These analyses revealed an age-dependent increase in all three *let-7-C* miRNAs in both adult males and females, suggesting a role for this miRNA cluster in aging-related processes.

To characterize the role of this age-dependent increase in *let-7-C* miRNAs, we analyzed a *let-7-C* hypomorphic (*let-7-C*^*hyp*^) strain in which *let-7-C* miRNAs were expressed during development [37] but not maintained during adulthood (Fig 1B, right). This hypomorphic strain was *trans*-heterozygous for two *let-7-C* null alleles but also harbored a single copy of a minimal *let-7-C* rescuing transgene that contained regulatory elements required for onset of *pri-let-7-C* (*let-7-Cp*^*3*.*3kb*^::*cDNA*) during development but lacked elements needed for its post-developmental maintenance. Consistent with our previous analysis [37], young *let-7-C*^*hyp*^ mutant males expressed reduced levels of *miR-100* (9.2±4.6% of control), *let-7* (22±10.2% of control) and *miR-125* (33±7.2% of control) that decreased further as the adults aged (Fig 1B). We therefore performed survival analysis of these *let-7-C*^*hyp*^ mutant males and found that they died prematurely relative to control males (Fig 1C, compare black and red curves; *w*^*1118*^: median survival 74d, maximum lifespan 98d; *let-7-C*^*hyp*^: median survival 36d, maximum lifespan 56d). Prompted by this reduced viability, we assayed the *let-7-C*^*hyp*^ strain for additional functional and morphological age-dependent phenotypes. Young *let-7-C*^*hyp*^ mutants climbed normally, indicating that the levels of *let-7-C* miRNAs they express during metamorphosis and early adulthood is sufficient for general adult function. However, aged *let-7-C*^*hyp*^ mutants displayed a steep reduction in this ability (Fig 1D). These results indicated that persistent expression of one or more of the three *let-7-C* miRNAs specifically during adulthood was required for normal adult healthspan.

Given the neural expression of *let-7-C* miRNAs [33, 37, 38], we next looked for age-associated deterioration in brain morphology. Brain degeneration has been anatomically characterized by an age-dependent increase in the number of scattered vacuoles that mark cells undergoing necrotic cell death [39]. Sections of 40-day old control and *let-7-C*^*hyp*^ brains revealed a sharp increase in vacuole number in mutant brains (Fig 1E–1G). As with the climbing defect described above, this phenotype had an adult onset since the brains of young mutant flies contained hardly any vacuoles (0 vacuoles in *w*^*1118*^, 0.6 ± 0.9 vacuoles in *let-7-C*^*hyp*^, n = 5). Importantly, a *let-7-C* transgene that substantially restored *miR-100* levels (59.4±11.1% of control), *let-7* levels (50.3±24% of control) and *miR-125* levels (108±28%) in 3-day old adults (*let-7-C*^*hyp*^ rescue in Fig 1B) rescued the lifespan and age-dependent climbing defects as well as the brain deterioration of *let-7-C*^*hyp*^ mutants (Fig 1C, 1D and 1G). Since our qRT-PCR analysis indicated that rescued *let-7-C*^*hyp*^ mutants express a constant level of *let-7-C* miRNAs during adulthood (Fig 1B), we inferred that the age-dependent increase in *let-7-C* miRNAs detected in wildtype adults was not absolutely required for their pro-survival and neuroprotective roles. Taken together, these results confirmed a role for *let-7-C* miRNAs in the aging processes that occur in the brain.

### *miR-125* and *let-7* mutants display reduced lifespan and neurodegeneration

In order to distinguish the roles of the three *let-7-C* miRNAs, we generated a set of rescuing transgenes with either *miR-100*, *let-7* or *miR-125* deleted. These transgenes were inserted into identical chromosomal locations using phiC31-mediated integration [40] and crossed into a *trans*-heterozygous *let-7-C* null background, yielding strains we referred to as *ΔmiR-100*, *Δlet-7* and *ΔmiR-125* single mutants, respectively (see S1 Fig for our crossing scheme that ensured that single mutant strains were otherwise as close to identical as possible). Unlike previously generated strains with P-element rescue transgenes [33], differences between these single mutants could be attributed to loss of an individual miRNA rather than to position effects.

**Fig 2:**
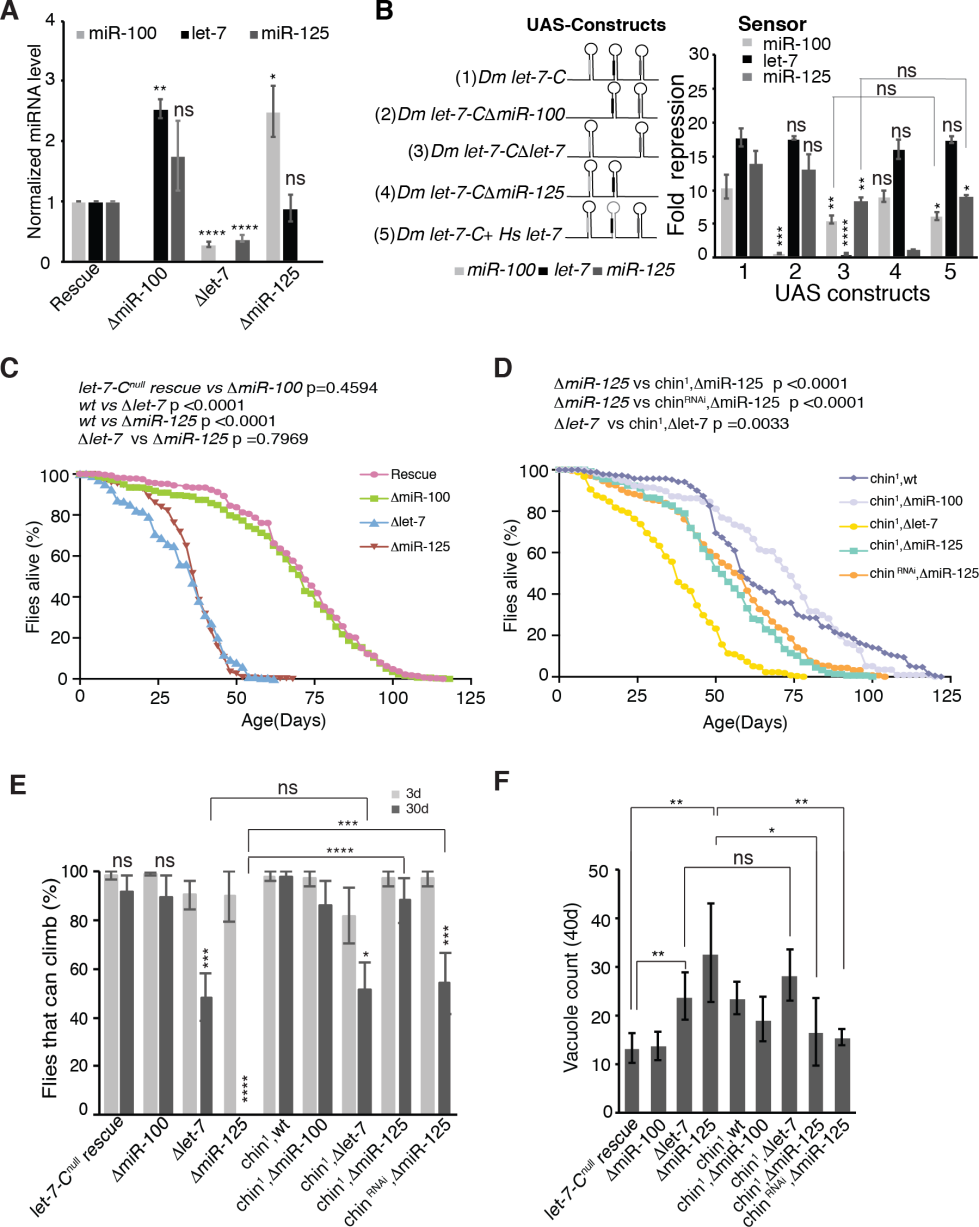
*ΔmiR-125* mutant aging phenotypes are suppressed by loss of *chinmo*. (**A**) Quantitative RT-PCR analysis of *miR-100*, *let-7* and *miR-125* in 3d old *ΔmiR-100*, *Δlet-7* and *ΔmiR-125* and rescue adult males. MiRNA levels were normalized to 2S rRNA. P values: ** < 0.01; **** < 0.0001. (**B**) Fold repression of *miR-100*, *let-7* and *miR-125* luciferase sensors in Kc-167 cells transfected with *UAS-let-7-C* constructs (schematic on left). Assays were performed in triplicate and the results were represented as Mean ± S.D. The data was statistically analyzed by an unpaired t-test. (**C**) *Δlet-7* and *ΔmiR-125* mutant males have a shortened life span (*let-7-C*^*null*^ *rescue*: median survival 72d, maximum lifespan 114d, n = 272; *ΔmiR-100*: median 76d, maximum lifespan 118d, n = 317; *Δlet-7*: median 36d, maximum lifespan 62d, n = 202; *ΔmiR-125*: median 38d, maximum lifespan 68d, n = 337 p values were calculated by Log-rank (Mantel-Cox) test. (**D**) Reducing one copy of *chinmo* or knock down of *chinmo* by RNAi rescue the lifespan defects of *ΔmiR-125* mutant but not *Δlet-7* mutant males (*chin*^*1*^*; wt*: median survival 58d, maximum lifespan 118d, n = 205; *chin*^*1*^*; ΔmiR-100*: median 74d, maximum survival 120d, n = 117; *chin*^*1*^*; Δlet-7*: median 38d, maximum survival 66d, n = 253; *chin*^*1*^*; ΔmiR-125*: median survival 52d, maximum lifespan 100d, n = 157; *chinmo RNAi/ΔmiR-125*: median survival 56d, maximum lifespan 104d, n = 281. P value of the lifespan curves was calculated by log-rank test. (**E**) *Δlet-7 and ΔmiR-125* mutant males display age associated climbing defects. At 3 days, the climbing ability of *Δlet-7* and *ΔmiR-125* males was comparable to the control flies expressing the wild type *let-7-C* transgene. However, at 30 days, only 48.29 ± 9.6% of *Δlet-7* flies and 0% of the *ΔmiR-125* flies were able to climb as opposed to 91.5 ± 6.7% of control flies. The age related climbing defects of *ΔmiR-125* and not *Δlet-7* was partially rescued by reducing *chinmo* levels. When aged for 30 days, 0% of *ΔmiR-125* flies, 88.1 ± 9.1% of *chin*^*1*^*; ΔmiR-125* flies, and 54.1± 12.5% of *chinmo RNAi/ ΔmiR-125* flies display climbing ability upon aging. However, aged *chin*^*1*^*; Δlet-7* flies displayed only a very slight increase in climbing ability (51. 6 ± 10.8%) when compared to age-matched *Δlet-7* flies (48.29 ± 9.6%). The data were statistically analyzed by an unpaired t-test. The results represented the mean ± S.D of three experiments, n = 15 male flies in each experiment, *** = p value <0.001. (**F**) *ΔmiR-125* and *Δlet-7* mutants display late onset brain degeneration that is rescued by reducing *chinmo* levels. Aged *ΔmiR-125* and *Δlet-7* males showed an increased number of vacuoles (relative to *let-7-C*^*null*^ *rescue*). Reduced dosage of *chinmo* in *ΔmiR-125* mutants decreased the vacuole count (Two tailed t-test, mean ± S.D n = 5). Genotypes used were the same as those listed for Fig 2A, 2C and 2D in S1 Table.

Quantitative RT-PCR analysis of *miR-100*, *let-7* and *miR-125* confirmed the absence of miRNA expression in each of the deletion lines (Fig 2A). However, this analysis also revealed cross-regulatory relationships between the three miRNAs: loss of *let-7* resulted in reduced levels of both *miR-100* (0.29 fold relative to control) and *miR-125* (0.35 fold relative to control), while loss of *miR-100* and *miR-125* resulted in increased levels of *let-7* (2.5 fold relative to control) and *miR-100* (2.5 fold relative to control), respectively (Fig 2A). To assess the cause of these changes, we turned to a cell culture assay in which we could quantify the activity of each miRNA in cells transfected with altered *let-7-C* versions. MiRNA activity was quantified as the fold repression in luciferase levels produced by previously validated “sensors” for each *let-7-C* miRNA [38]. Individual sensors were co-transfected along with UAS-*let-7-C* cDNA constructs into Kc-167 cells that do not ordinarily express *let-7-C* miRNAs [37]. First, confirming the effect of *let-7* deletion on *miR-100* and *miR-125* levels, we found that *miR-100* and *miR-125* activity reporters were less repressed in cells transfected with a *let-7-C* cDNA lacking the *let-7* hairpin (miR-100: 5.6 ± 0.56 fold repression in *Δlet-7* compared to 10.48 ± 1.7 in wild type; *miR-*125: 8.3 ± 0.66 fold in *Δlet-7* compared to 13.8 ± 1.97 fold in *wild type*). Then, to test whether this effect was due to the absence of mature *let-7* or some other cause (e.g. altered RNA conformation of the *Δlet-7* primary transcript that reduced *miR-100* and *miR*-*125* processing), we generated a chimeric *UAS let-7-C* cDNA construct in which the *Drosophila let-7* hairpin was replaced with the human *let-7-a2* hairpin that encoded the same mature *let-7* but has a different hairpin structure. While the human *let-7-a2* hairpin restored the *let-7* mediated repression of its sensor, it did not restore *miR-100* and *miR-125* mediated repression (Fig 2B, construct 5). These data indicated that processed *let-7* miRNA did not directly regulate the processing of *miR-100* or *miR-125*. Instead, we favor a model where the rate of *let-7* processing has an effect on the rate of *miR-100* and *miR-125* processing, a model consistent with processing of other polycistronic microRNAs [41]. We note that Truscott et al. also recently found evidence for cross-regulatory interaction between *let-7-C* miRNAs [41], although their results were slightly different—deletion of *let-7* and *miR-100* but not *let-7* alone reduced *miR-125* levels—probably due to technical differences in the constructs used. We also evaluated *ΔmiR-100* or *ΔmiR-125 let-7-C* cDNA constructs in this cell culture assay, but detected no enhancement in miRNA activity (Fig 2B), suggesting that the changes in miRNA levels detected in tissue (Fig 2A) may not be functionally significant. Taken together, these results indicated that the set of *ΔmiR-100*, *Δlet-7* and *ΔmiR-125* strains described above would allow the dissection of the individual contributions of the three miRNAs since neighboring miRNAs continued to be expressed when individual miRNAs were deleted, albeit at altered levels in some cases.

We then used the *ΔmiR-100*, *Δlet-7* and *ΔmiR-125* single mutant lines to analyze the consequences of deleting each miRNA on age-associated brain degeneration and behavioral defects. *Δlet-7* and *ΔmiR-125* single mutant flies displayed significantly reduced longevity compared to control or *ΔmiR-100* flies (Fig 2C). In addition, while young *Δlet-7* and *ΔmiR-125* mutants had normal climbing behavior and brain morphology, a significant decrease in climbing ability as well as a marked increase in vacuole number was observed in both mutants with age (Fig 2E and 2F). The vacuoles in both *Δlet-7* and *ΔmiR-125* mutants appeared to be scattered throughout the central brain region and some enrichment was also seen in the retina (S2 Fig). These data indicated that loss of either *let-7* or *miR-125* but not *miR-100* caused behavioral and morphological changes that were normally seen in much older flies and were indicative of rapid aging of the brain.

### *miR-125* and *let-7* enhance neurodegeneration at distinct stages during the life cycle

Given that loss of *let-7* and *miR-125* triggered physiological processes involved in aging, we tested whether inhibition of individual *let-7-C* miRNAs enhanced the neurodegeneration of a disease model of fragile X-associated tremor/ataxia syndrome (FXTAS) [42]. FXTAS is a late onset human neurodegenerative disease that is characterized by the presence of ubiquitin positive nuclear inclusions containing RNAs with expanded CGG repeats (rCGG) in neurons and astrocytes [43]. Ectopic expression of transcripts with artificial expansion of these repeats in the fly retina causes a pathology similar to human FXTAS, including photoreceptor degeneration and disorganization of the ommatidia [42]. Using miRNA “sponge” constructs designed to individually inhibit *miR-*100, *let-7*, or *miR-125* (*miR-100SP*, *let-7SP*, or *miR-125SP*), we tested whether loss of any of these miRNAs’ activities enhanced the retinal degeneration in the FXTAS model. We found that driving *let-7SP* or *miR-125SP* but not *miR-100SP* specifically in the eye throughout development and adulthood resulted in significant enhancement of the rCGG phenotype (S3 Fig). This result indicated that, in addition to their role in modulating lifespan, *let-7* and *miR-125* promoted disease pathogenesis while *miR-*100 did not.

**Fig 3:**
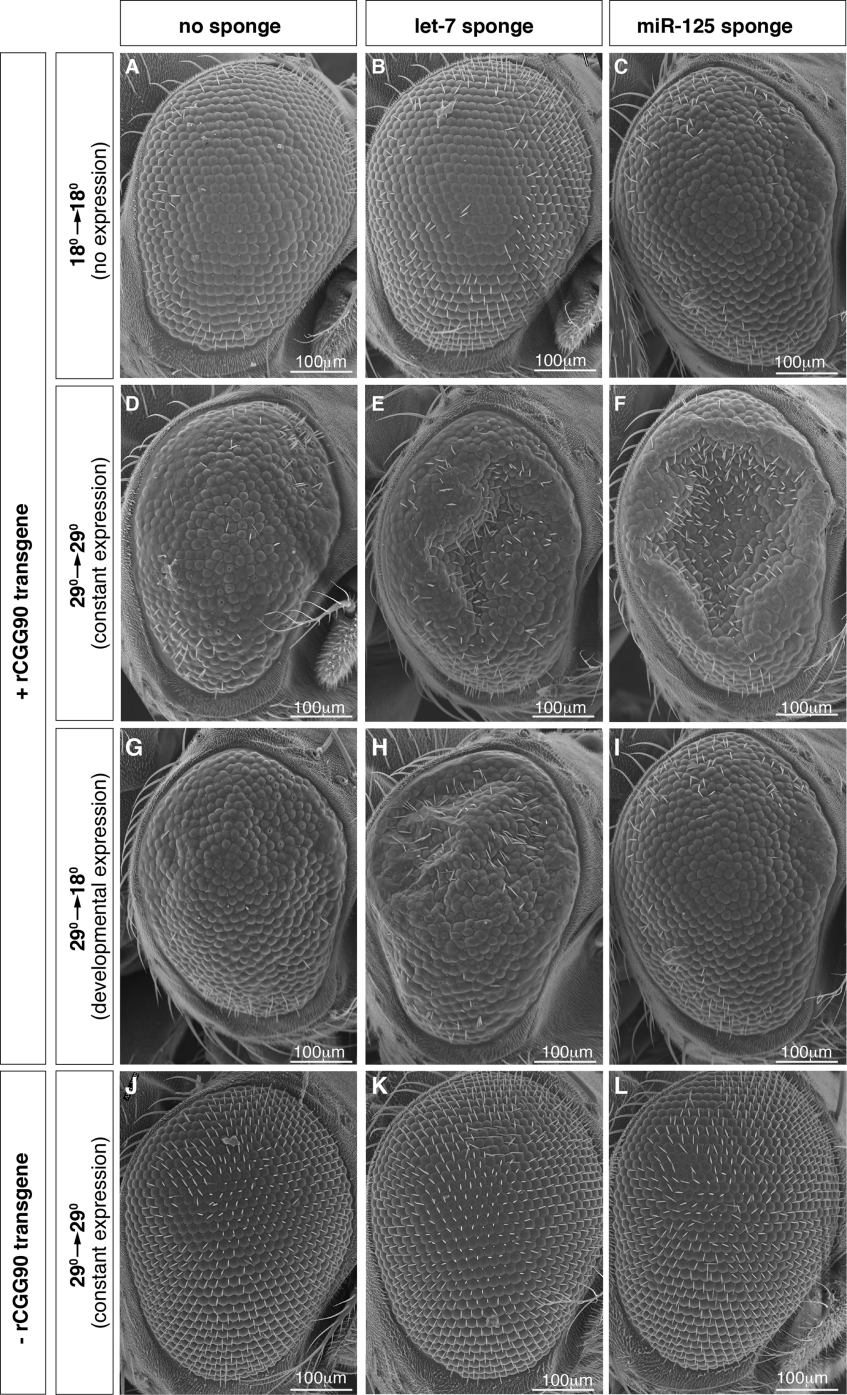
Loss of *miR-125* and *let-7* enhance rCGG_90_ mediated retinal degeneration during different stages of the life cycle. Scanning electron microscope eye sections from 10 day old flies harboring a *GMR-Gal4* transgene (all panels), a *tubP-Gal80*^*ts*^ transgene (all panels), a *UAS-rCGG*_*90*_ transgene (**A-I**), a *UAS-let-7SP* sponge transgene (**B**, **E**, **H**, **K**), and/or a *UAS-miR-125SP* sponge transgene (**C**, **F**, **I**, **K**) and reared under one of three conditions: 18°C during development and adulthood (18→18, panels **A-C**), 29°C during development and adulthood (29→29, panels **D**-**F** and **J**-**L**), or 29°C during development and 18°C during adulthood (29→18, panels **G**-**I**). Scale bars, 100μm.

To pinpoint the specific stage during which *let-7* and *miR-125* activity were involved in FXTAS disease pathogenesis, we utilized a temperature sensitive allele of Gal80 (*tubP-Gal80*^*ts*^). This approach allowed temporal control of both the *UAS-rCGG*_*90*_ transgene as well as the *UAS-miRNA* sponges in the eye, since animals at 29°C express UAS transgenes but animals at 18°C do not [44]. We reared strains to control expression in three ways: no expression (18→18), constant expression (29→29), or expression during development but not adulthood (29→18) (Fig 3A–3L). As expected, constant expression of either *let-7SP* or *miR-125SP* enhanced the rCGG_90_ phenotype whereas no expression did not (Fig 3A–3F). However, *let-7SP* and *miR-125SP* behaved differently from one another in the 29→18 regimen: *let-7SP* enhanced rCGG_90_ retinal degeneration while *miR-125SP* did not (compare Fig 3G–3I). This result, along with the observation that *let-7SP* animals reared at 29→18 looked no worse than those reared at 29→29, suggested that *let-7*’s main contribution to disease progression occurred during development. Conversely, *miR-125SP* animals reared at 29→18 looked no worse than those reared at 18→18, suggesting that the phenotypes displayed by those reared at 29→29 was a specific consequence of adult *miR-125SP* expression. The reciprocal experiment involving adult-only transgene expression was not informative because none of the 18→29 animals displayed a phenotype, even when aged up to 20 days, perhaps because the underlying rCGG_90_ phenotype was at least partially of developmental origin. These data indicated that *let-7* and *mir-125* functioned during distinct temporal periods to effect disease progression, and that the retinal degeneration caused by a decline in *let-7* activity was of a developmental origin while that caused by inhibition in *miR-125* activity was due to degeneration of adult brains.

### *miR-125* regulates aging *via* repression of *chinmo*

Since miRNAs function by repressing target mRNAs, we investigated whether the age-associated *Δlet-7* and *ΔmiR-125* phenotypes were due to the elevated expression of *chronologically inappropriate morphogenesis* (*chinmo*), the only verified target of both *let-7* and *miR-125* in flies [38]. Chinmo is a transcription factor that controls neuronal fate in a dosage-sensitive manner. In the mushroom body lineages in the central brain, for example, Chinmo is expressed at high levels early in development to promote early born cell fates and its post-transcriptional downregulation leads to the production of later born fates [45]. The *chinmo* 3’UTR contains multiple *let-7* and *miR-125* binding sites and is regulated by *let-7-C* miRNAs during development [38]. To address whether the adult phenotypes of *Δlet-7* and *ΔmiR-125* mutants were due to elevated Chinmo, we reduced the dosage of *chinmo* in these mutants by either removing one copy of *chinmo* using a null *chinmo*^*1*^ mutation [45] or by knocking down *chinmo* using a RNAi transgene that we verified *in vivo* (S4A–S4L Fig). Lowering *chinmo* levels dramatically suppressed the premature death (Fig 2D), climbing defects (Fig 2E), and brain necrosis (Fig 2F and S4M Fig) of *ΔmiR-125* mutants but not *Δlet-7* mutants. This result indicated that elevated Chinmo was responsible for *ΔmiR-125* phenotypes but that other factors were responsible for *Δlet-7* phenotypes. This distinction also indicated that the *Δlet-7* phenotypes described above (reduced longevity, climbing and neurodegeneration) were not solely due to the reduction in *miR-125* levels observed in these mutants (Fig 2A) and implied the de-repression of other, currently unidentified, mRNAs.

**Fig 4:**
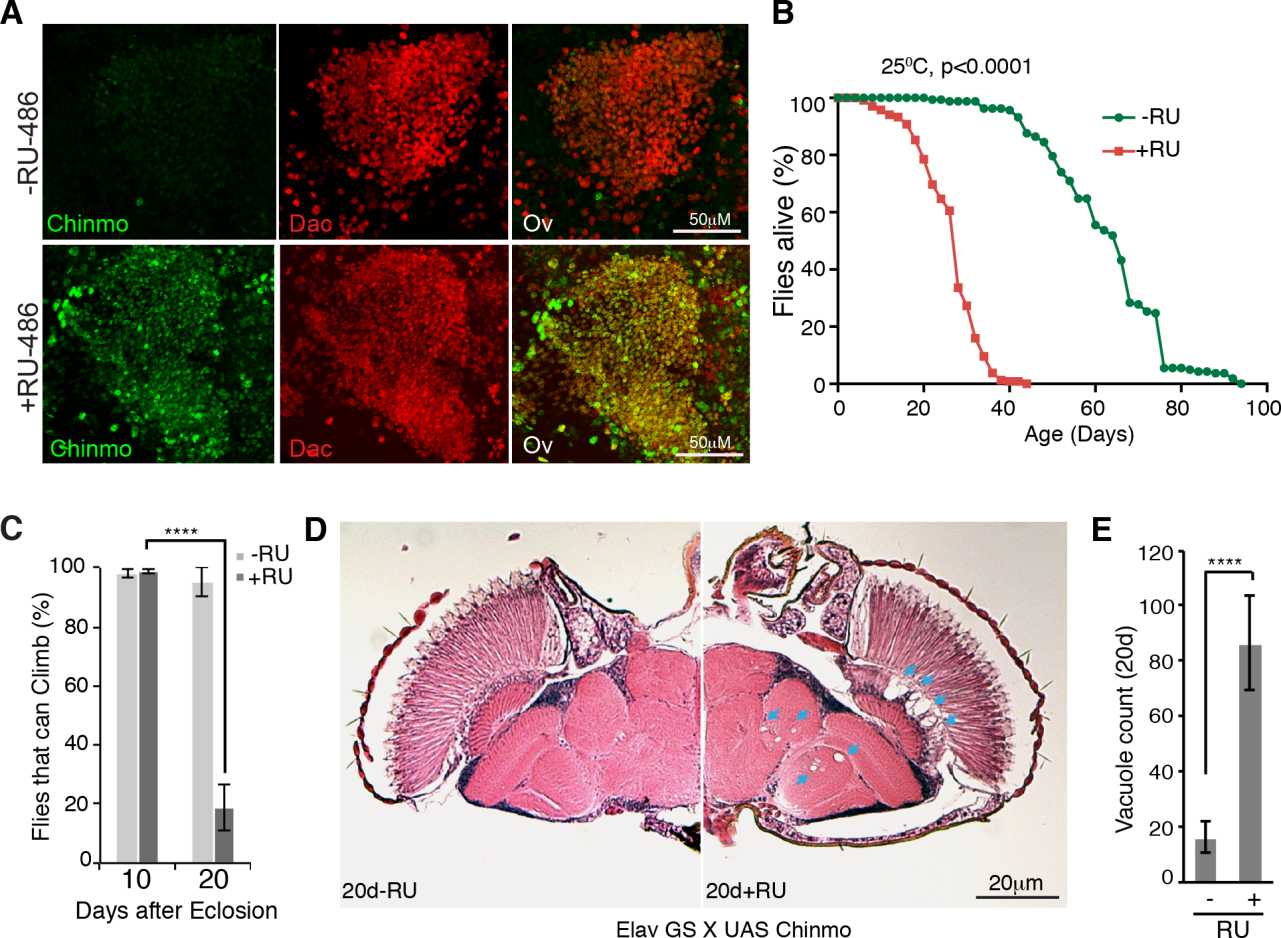
Overexpression of Chinmo in adult neurons reduces adult lifespan and healthspan. *UAS-chinmo* was driven in the adult nervous system in a drug inducible manner with a neural Gene-Switch Gal4 driver (*3XelavGS*). (**A**) RU-486-fed males displayed elevated neural Chinmo levels, as detected by Chinmo (green) and Dachshund (red) staining of the brains of *UAS-chinmo* / *3XelavGS* adult flies that were fed either 200μM RU-486 (+RU) or ethanol (-RU) for 5 days. (**B**) RU-486-fed males (+RU) displayed reduced lifespan relative to controls (-RU) (+RU-486: median survival 28d, maximum lifespan 44d, n = 238 males; *-*RU-486 [control]: median survival 66d, maximum lifespan 94d, n = 162 males). P value of the lifespan curves was calculated by log-rank test. (**C**) RU-486-fed males (+RU) displayed age-associated climbing defects. While normal at 10 days, only 18.8 ± 7.9% of RU-486 fed flies (+RU) were able to climb as opposed to 95 ± 5% of control flies (-RU) at 20 days. Mean ± S.D of three experiments, n = 15 male flies, *** = p value <0.001, two tailed t-test. (**D-E**) Brain sections of 20d RU-486-fed males (+RU) displayed elevated numbers of vacuoles than controls (-RU) (+RU, 86.4 ± 16.9 vacuoles per brain; -RU, 16.2 ± 5.5 vacuoles per brain, p<0.0001, two tailed t-test, mean ± S.D, n = 5). Arrows in D point at vacuoles. The genotype of all flies used in this figure is listed in S1 Table.

To test whether ectopic Chinmo was sufficient to cause the neurodegenerative phenotypes associated with loss of *miR-125*, we ectopically expressed a *chinmo* transgene in adult brains using an inducible neural GAL4 driver (Fig 4A). This forced expression resulted in a drastic reduction in both lifespan (Fig 4B) and climbing ability (Fig 4C) along with a dramatic increase in brain vacuole numbers in 20d aged flies (Fig 4D and 4E). Interestingly over- expression of Chinmo in neurons showed an increased localization of vacuoles in the lamina and central brain regions, indicating that neurons in the lamina region were more sensitive to de-regulation of Chinmo (Fig 4D and 4E and S2E Fig). These experiments confirmed that deregulated expression of Chinmo in adult neurons results in premature neurodegeneration in adult flies and supported the genetic suppression evidence above that silencing of this protein by *miR-125* was critical for maintaining neuronal integrity and viability in adult flies.

### Biphasic regulation of *chinmo* by *let-7* and *miR-125*

**Fig 5:**
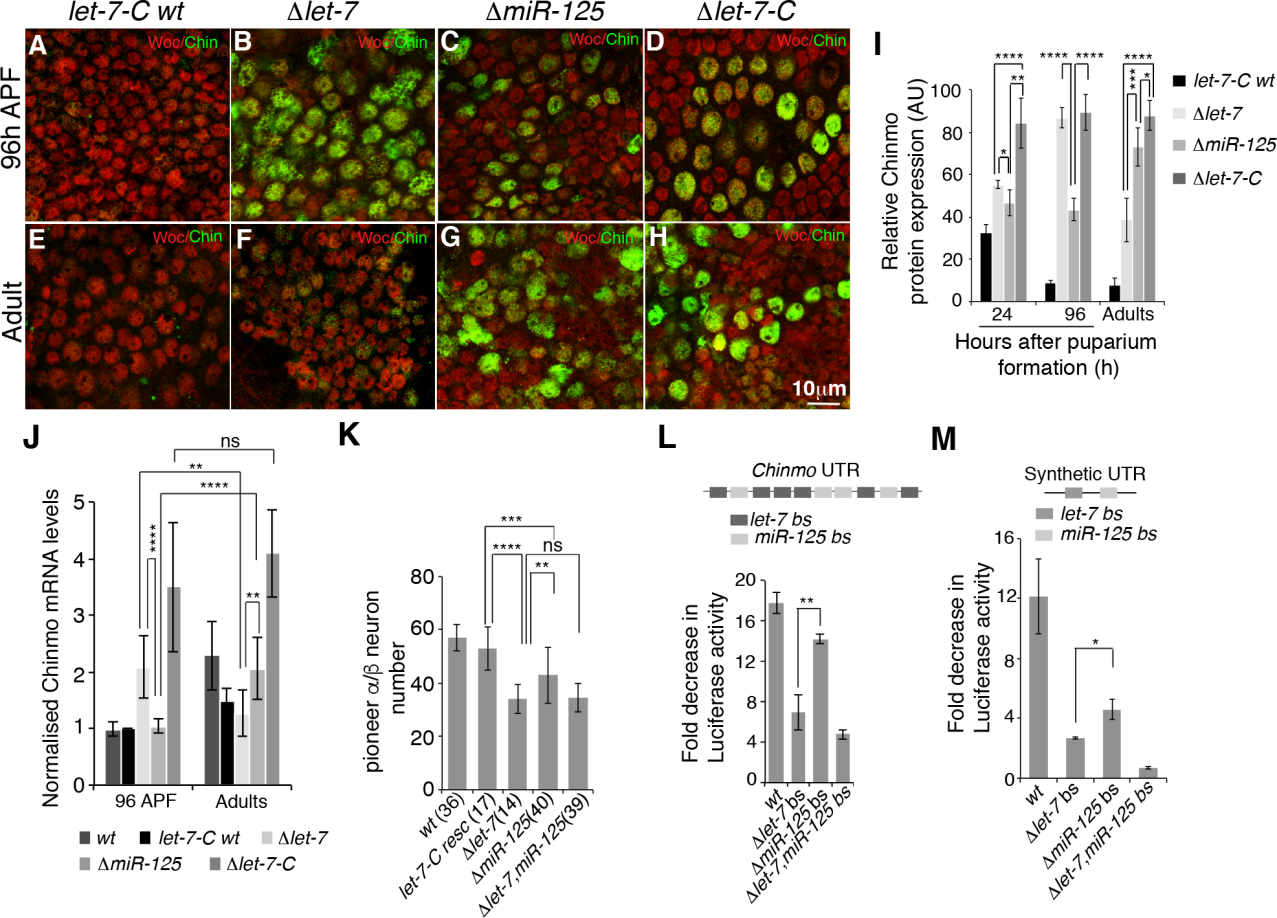
A *let-7*-to-*miR-125* switch represses Chinmo in adult flies. (**A**-**H**) Single confocal sections of late pupal (96h APF) and young adult (Adult) brains dissected from *Δlet-7*, *ΔmiR-125* and *ΔmiR-100*, *let-7*, *miR-125* and rescued animals stained for Chinmo (green) and Without Children (Woc, red). (**I**) Chinmo protein levels in *Δlet-7*, *ΔmiR-125*, and *Δlet-7-C* brains at indicated time points. (**J**) *chinmo* mRNA levels in *Δlet-7*, *Δmir-125* and *Δlet-7-C* mutant heads, normalized to *kinesin*. (**K**) Quantification of *c708a*-positive neuron number in adult brains of indicated genotypes (numbers analyzed in parentheses). See S1 Table for detailed genotypes. Data represent mean ± S.D (**I**, **J**, **K)**. (**L**, **M**) *let-7* exhibits a significantly higher degree of post-transcriptional repression than *miR-125*. (**L**) Fold repression of luciferase reporters containing a 1.4kb *chinmo* 3‘UTR and mutated 1.4kb 3‘UTR fragments lacking either *let-7* or *miR-125* sites in Kc-167 cells in which *let-7-C* was ectopically expressed. (**M**) Fold repression of synthetic luciferase reporters having a single *let-7* and *miR-125* miRNA binding sites. See S5 Fig for sequence information. Values represented as mean ± S.D.

Messenger RNAs frequently contain binding sites for multiple miRNAs, which may repress common targets in an additive manner [31]. However, our analysis raised the possibility that the *chinmo* mRNA might be regulated by *miR-125* but not *let-7* in adult brains despite containing verified functional binding sites for *let-7* [38]. Intriguingly, these experiments were supported by immunostaining of Chinmo in *Δlet-7* and *ΔmiR-125* adult brains. The degree of de-repression of Chinmo was more widespread and starkly higher in *ΔmiR-125* brains when compared to *Δlet-7* (S5D Fig). In contrast, *Δlet-7* mutant brains displayed a much weaker immunostaining signal of Chinmo (S5C Fig). To distinguish the contributions of *let-7* and *miR-125* on *chinmo* repression, we compared the levels of Chinmo protein in immunostained brains of control, *Δlet-7*, *ΔmiR-125*, and *let-7-C* null adults. Shortly after *let-7-C* activation at 24 hours after puparium formation (APF), elimination of both miRNAs resulted in much higher levels of Chinmo than loss of either miRNA alone (Fig 5A–5I), indicating that both *let-7* and *miR-125* contributed to *chinmo* repression at this time-point. Three days later, however, *let-7* played the predominant role in silencing *chinmo*, since *Δlet-7* mutant brains expressed 86.7 ± 4.8 arbitrary units (AU) of Chinmo while *ΔmiR-125* mutant brains expressed only 43.3 ± 5.2 AUs of Chinmo (Fig 5A–5D and Fig 5I). A complete reversal in the relative contributions of *let-7* and *miR-125* occurred during the pupal-to-adult transition: *Δlet-7* mutant adult brains expressed 38.5 ± 10.3 AUs while *ΔmiR-125* mutant brains expressed 72.9 ± 9.2 AUs, indicating that *miR-125* was primarily responsible for silencing *chinmo* in adults (Fig 5E–5I and S5A–S5D Fig). To support this data, we also quantified the levels of *chinmo* mRNA in late pupal and adult *Δlet-7* and *ΔmiR-125* mutant heads, since miRNA regulation is known to cause mRNA destabilization. Consistent with our quantification of Chinmo protein levels, loss of *let-7* affected *chinmo* mRNA levels in late pupae but not adults, while loss of *miR-125* affected *chinmo* mRNA levels in adults but not pupae (Fig 5J). Together, these data indicated that *let-7* and *miR-125* predominantly regulated *chinmo* during development and adulthood, respectively.

Since the derepression of Chinmo during development was higher in *Δlet-*7 than *ΔmiR-125* mutants, we tested whether *chinmo*-dependent developmental defects were more severe in *Δlet-*7 than *ΔmiR-125* single mutants. To examine this possibility, we investigated the relative roles of *let-7* and *miR-125* in fate transitions in MB neuronal temporal identity. The MB is composed of four subtypes of neurons that are generated in a sequential *manner* (γ → α’/β’ → pioneer α/β → α/β) by neuroblasts [45–47]. High levels of Chinmo specify early born cell fates (γ, α’/β’), while low levels of Chinmo specify later born cell fates (pioneer α/β, α/β). Altered dosages of *chinmo* lead to changes in the total numbers of these various neuronal classes so that, for example, elevated *chinmo* is associated with a smaller population of pioneer α/β neurons [38, 45]. Therefore, to evaluate the relative contributions of *let-7* and *miR-125 chinmo* regulation during neural development, we counted the number of pioneer α/β neurons in adult brains. As expected, *Δlet-7* mutants displayed greater reduction in pioneer α/β neuron number than *ΔmiR-125* mutants and, furthermore, *Δlet-7*, *miR-125* double mutants showed a reduction similar to *Δlet-7* single mutants (Fig 5K). Thus, regulation of *chinmo* was more dependent on *let-7* than *miR-125* during the larval-to-adult transition but was more dependent on *miR-125* in aging adults.

To assess whether *let-7* and *miR-125* had different strengths of repression that might contribute to their differential activities, we examined the expression of luciferase reporters containing a previously characterized 1.4kb 3’UTR fragment of *chinmo* that harbors six *let-7* and four *miR-125* binding sites that are conserved between *Drosophila* species [38]. Overexpression of the entire *let-7-C* primary transcript in Kc-167 cells repressed the wild type reporter 17-fold (Fig 5L). In contrast the fold repression of mutants lacking *let-7* binding sites or *miR-125* sites or both was reduced 6.9-, 14.0- and 4.8-fold, respectively. While these data indicated that *let-7* was a stronger repressor of *chinmo* than *miR-125*, it was not clear whether the greater decrease in fold repression was due to a greater number of *let-7* sites. To circumvent this issue, we designed and quantified the degree of repression of a luciferase reporter that contained a single verified miRNA binding site for *let-7* and a single *miR-125* site that had comparable base pairing characteristics (S6 Fig). Ectopic expression of *let-7-C* miRNAs resulted in a 12-fold repression of the wild type reporter while deletion of the *let-7* seed sequence, the *miR-125* seed sequence or both sequences resulted in a 2.9-, 4.6- and 0.7-fold repression of luciferase activity respectively (Fig 5M). Together these data confirmed that both *let-7* and *miR-125* were capable of silencing the luciferase sensor individually but maximum repression was achieved when both sites were functional and that *let-7* was a stronger post-transcriptional repressor than *miR-125*.

### Differential processing and turnover rates of *let-7* and *miR-125* direct a switch in miRNA targeting activity during the larval-to-adult transition

**Fig 6:**
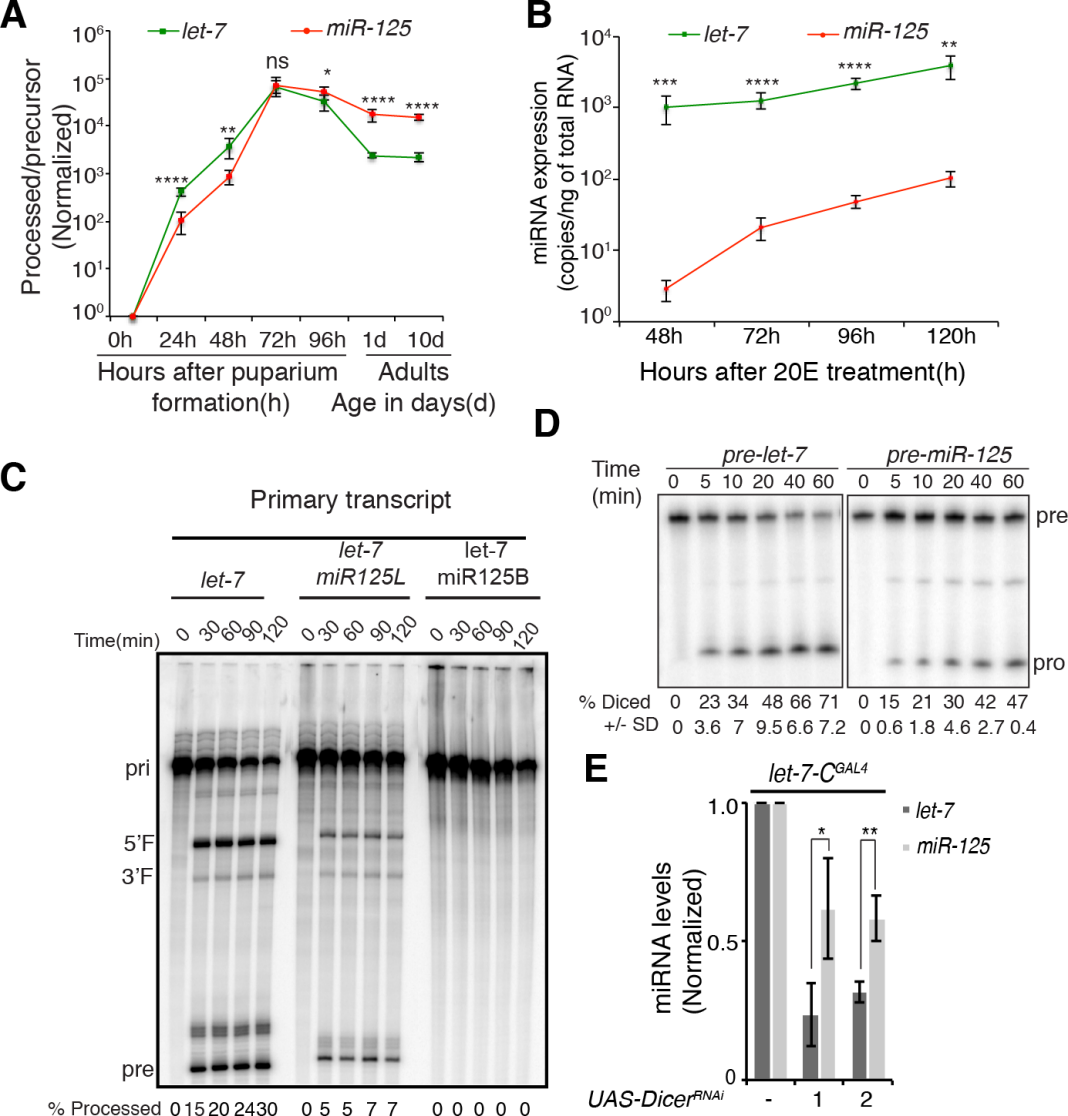
Differential rates of processing and turn over direct the *let-7*-to-*miR-125* switch during the larval-to-adult transition. (**A**) *mir-125* persists longer in adult brains. The developmental profile of precursor and processed *let-7* and *mir-125* as determined by qRT-PCR of total RNA extracted from dissected CNS of pupae and adults. Quantitation of processed/precursor miRNAs indicates that persistence of *miR-125* increases after 72h APF and exceeds that of *let-7* in adult brains. (**B**-**D**) *let-7* is processed more efficiently than *miR-125*. (**B**) Absolute levels of *let-7* and *miR-125* expression in 20-hydroxyecdysone (20E) treated Kc-167 cells. Total RNA was extracted from indicated time points after Kc-167 cells were treated with 20E (5μM) for 24h. To derive absolute levels of expression, a standard curve was generated using synthetic *let-7* and *miR-125* RNA oligonucleotides and CT values were extrapolated from the curves. Quantitation of copy number per nanogram of total RNA indicated a significantly higher processing rate of *let-7*. Values represented as mean of three experiments ± S.D. (**C**) Primary *let-7* transcripts expressing either *miR-125* terminal loop (*pri-let-7*^*miR-125L*^) or stem base (*pri-let-7*^*miR-125B*^) displayed slower kinetics of Drosha processing than the wild type *pri-let-7* transcript. *In vitro* processing of *pri-let-7*, *pri-let-7*^*miR-125B*^ and *pri-let-7*^*miR-125L*^ with purified Flag tagged Drosha-Pasha complex. The primary transcript (pri), 5’ flank (5’F), 3’ flank (3’F) and precursor (pre) are indicated on the left. Quantitation of the fraction processed is calculated as precursor/primary +5’F+3’F+precursor. (**D**) *Pre-let-7* is diced more efficiently than *pre-miR-125*. *In vitro* processing of radiolabeled *pre-let-7* and *pre-miR-125* with purified Flag-tagged Dicer 1. Products were analyzed on a 10% polyacrylamide gel. The precursor and the processed miRNA are indicated on the left. Quantitation of fraction diced is indicated at the bottom of the gel. (**E**) *let-7* has a higher turnover rate than *miR-125* in the adult central nervous system. Expression levels of *let-7* (dark gray) and *miR-125* (light gray) in adult fly heads of lines expressing *Dicer-1* RNAi transgenes under the control of *let-7-C*-*Gal4* as determined by Taqman qRT-PCR. (**A**, **E**) Values represented as mean of three experiments ± S.D. 2S rRNA was used as normalization control.

To investigate the basis for the sequential repression of Chinmo by *let-7* and *miR-125*, we first compared the rate of *let-7* and *miR-125* production in the developing and adult nervous system. To do so, we quantified the ratio of processed miRNA to precursor miRNA for *let-7* and *miR-125* in staged nervous system samples (Fig 6A). The *let-7/pre-let-7* ratio was significantly higher than the *miR-125/pre-miR-125* prior to 72h APF. In contrast, the *miR-125/pre*-*miR-125* ratios were higher than the *let-7/pre-let-7* ratios after 72h APF and into adulthood (Fig 6A). The temporal dynamics of *let-7* and *miR-125* production in the nervous system correlated with their relative roles in *chinmo* regulation, suggesting that the basis for their sequential activity may involve differential processing and/or turnover.

To determine the basis for this switch in the relative expression of *let-7* and *miR-125*, we first investigated the possibility that *let-7* and *miR-125* might be differentially processed. To do so, we again took advantage of the Kc-167 embryonic cell line. As mentioned above, the *let-7-C* locus is not ordinarily transcribed in this cell line, but it is activated in response to the *Drosophila* steroid hormone 20-hydroxyecdysone (20E) [37]. Kc-167 cells were treated with 20E for 24h to induce primary *let-7-C* transcript and the levels of *let-7* and *miR-125* were monitored at different time intervals after washing off the steroid hormone. To measure the relative rates of processed *let-7* and *miR-125* production, we performed qRT-PCR on 20E-treated Kc-167 samples using a standard curve to extrapolate absolute miRNA levels. Forty-eight hours after the 20E treatment, we detected 1024±440 copies of *let-7* per nanogram (ng) of total RNA but only 3±1 copies of *miR-125* per ng of total RNA, indicating that *let-7* was processed more efficiently than *miR-125* (Fig 6B). Incubation of cells for 120 hours after the 20E pulse resulted in a 3–4 fold increase in the copy number of *let-7* (3872±1365 copies/ng of total RNA) and a ~30 fold increase in the copy number of *miR-125* (101±24 copies/ng of total RNA). The greater increase in the *miR-125* copy number at later time points suggested that processed *miR-125* persisted longer than *let-7*.

In order to identify the key steps in miRNA biogenesis that contributed to the inefficient processing of *miR-125*, we performed *in vitro* Drosha and Dicer processing assays (Fig 6C and 6D). We first examined the rate of generation of precursor miRNAs (pre-miRNA) from longer primary miRNA (pri-miRNA) transcripts by the Drosha-Pasha complex. In initial experiments, we found that *pri-miR-125* processing was extremely inefficient (S7A Fig). Therefore, we compared the processing of *pri-let-7* to the processing of chimeric constructs in which either the *pri-let-7* terminal loop or its stem-base were replaced with the *pri-miR-125* loop (*pri-let-7*^*miR-125L*^) or *pri-miR-125* stem base (*pri-let-7*^*miR-125B*^), respectively (Fig 6B and S7B Fig). The rates of processing of these three transcripts were examined by incubating with Drosha-Pasha complexes immunoprecipitated from Kc-167 cells (S7B Fig). Substituting either the terminal loop or stem base of *pri-miR-125* in *pri-let-7* resulted in a dramatic reduction in Drosha processing. While 15% of the unmodified *let-7* primary transcript was processed within 30 minutes, only 5% of *pri-let-7*^*miR-125L*^ was cleaved by Drosha. Incubation with Drosha-Pasha complex for 120 minutes increased the percentage of precursor to 30% and 7% for *pri-let-7* and *pri-let-7*^*miR-125L*^, respectively. However, substituting the stem-base of *pri-miR-125* in *pri-let-7* completely abolished its Drosha processing (Fig 6C). Thus, both the terminal loop and stem base sequence determinants of *pri-miR-125* contributed to its inefficient processing by Drosha *in vitro*, raising the possibility that other post-transcriptional mechanisms may facilitate miR-125’s processing *in vivo*. To evaluate the Dicer-1 processing of *pre-let-7* and *pre-miR-125*, we also performed *in vitro* processing assays with Flag-tagged Dicer-1 that was, like the Drosha-Pasha complexes described above, purified from Kc-167 cell extracts (S7B Fig*)*. In these assays, *pre-miR-125* displayed a significantly lower kinetics of processing than *pre-let-7* (Fig 6C). Within 10 minutes of incubation with Dicer-1, 23±3.6% of *pre-let-7* and 15±0.6% of *pre-miR-125* were processed to their mature forms. After 60 minutes of incubation, the percentage diced was 71±7.2% and 47±0.4% for *pre-let-7* and *pre-miR-125*, respectively (Fig 6D and 6E). Thus, this higher kinetics of processing of *let-7* by both Drosha and Dicer likely contributed to its rapid accumulation during metamorphosis.

While differential processing was consistent with the more rapid accumulation of *let-7*, we hypothesized that the temporal dynamics of *miR-125* accumulation (Fig 6A) might also reflect an increased stability. In order to monitor the persistence of *let-7* and *miR-125* in adult nervous system tissue, we measured the expression of these miRNAs after blocking *Dicer-1* activity. Total RNA was extracted from heads of 10d-old adult flies that expressed one of two *Dicer-1* shRNA constructs, and the levels of *let-7* and *miR-125* were quantified by Taqman miRNA assays (Fig 6D and S7C Fig). Knockdown of *Dicer-1* resulted in a greater reduction of *let-7* relative to *miR-125* (Fig 6D): expression of *Dicer-1*^*shRNA1*^ or *Dicer-1*^*shRNA2*^ resulted in 0.23±0.1 or 0.31±0.37 fold expression of *let-7* relative to control but 0.62±0.18 or 0.58±0.18 fold expression of *miR-125* relative to control, respectively. These data indicated that the decay rate of *let-7* was significantly higher than that of *miR-125* in the adult nervous system.

**Fig 7:**
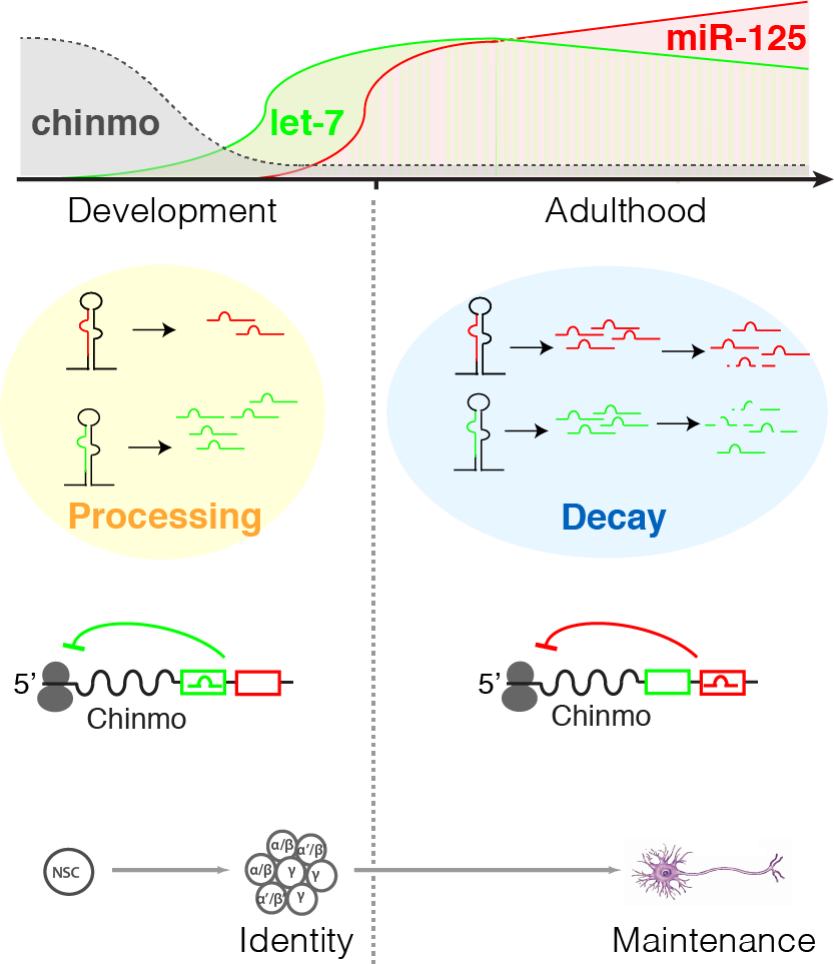
Biphasic action of two co-transcribed microRNAs. *Pre-let-7* and *pre-miR-125* are co-transcribed, but mature *let-7* accumulates more rapidly than mature *miR-125* during development due to its higher rate of processing by Drosha and Dicer. The enhanced stability of *miR-125* leads to a switch in the relative abundance of the two miRNAs during adulthood. This differential temporal expression contributes to the distinct functions of the two miRNAs during the life of a neuron: *let-7* fine-tunes the gradient of the dosage-sensitive transcription factor *chinmo* to control temporal cell fate determination during neural stem cell (NSC) division (for example, adjusting γ, α’/β’, and α/β identities in the mushroom body lineage) while *miR-125* ensures the complete silencing of *chinmo* during adulthood to promote neuron maintenance. Such phasic control may be a general feature of clustered miRNAs.

Finally, to assess whether *miR-125* had a longer half-life than *let-7*, we measured the decay rates and half-lives of *let-7* and *miR-125* by analyzing Kc-167 cells transfected with synthetic miRNA duplexes. Cells were washed with fresh medium 5 hours after transfection, and samples were collected at the indicated times for total RNA preparation followed by quantitation of *let-7* and *miR-125* by Taqman assays. Half-lives were inferred from fitted exponential curves (S7D Fig). As expected, the half-life of *miR-125* (T_1/2_, 2.7h) was significantly higher than that of *let-7* (T_1/2_, 2h). Taken together these experiments suggested a mechanistic basis for the switch in *let-7*-to-*miR-125* activity that occurred during pupal-to-adult transition: while an increased rate of Drosha/Pasha and Dicer processing of *let-7* facilitated the attenuation of *chinmo* in the developing nervous system, the enhanced perdurance of *miR-125* ensured that *chinmo* was silenced in the adult brains (Fig 7).

## Discussion

### Summary and model

*let-7* and *miR-125* have distinct and non-overlapping functions, despite being co-transcribed and sharing the same target. Loss of either miRNA alone leads to shortened lifespan and premature deterioration of health, as indicated by age-dependent climbing defects and brain degeneration. The aging defects caused specifically by loss of *miR-125* are associated with high levels of Chinmo in adult brains, and can be rescued by reducing *chinmo* levels in the *ΔmiR-125* mutant. In contrast, Chinmo is substantially lower in *Δlet-7* mutant adult brains and it appears not to contribute to adult *Δlet-7* mutant phenotypes: neither adult *Δlet-7* mutant climbing defects, brain vacuolization, nor reduced longevity are suppressed by *chinmo* reduction. Instead, *let-7* predominates during development: pupal Chinmo expression is higher and associated defects in neuronal identity are worse in *Δlet-7* mutants than *ΔmiR-125* mutants. Although deletion of *let-7* reduces *miR-125* levels, the differences in the *Δlet-7* and *ΔmiR-125* phenotypes indicate that the *Δlet-7* phenotypes are not simply due to loss of miR-125. In support of this, distinct temporal periods of *let-7* and *miR-125* activity were also identified using sponges, an independent method for disrupting miRNA activity in which *let-7* interference does not affect *miR-125* activity. Based on these results, we conclude that a *let-7*-to-*miR-125* switch during the pupal-to-adult transition ensures *chinmo* repression in adults, maintaining neuronal integrity and promoting life span.

Our results illuminate a function of miRNA co-targeting that we term “phasic control,” which indicates that co-targeting can reflect non-redundant regulation during distinct phases of a cells life, from its birth to its death. Rather than simply reinforcing silencing, such repression at different times may have distinct functions, based not only on the changing status of the cell but also on differences in miRNA::mRNA interactions (e.g. base-pairing characteristics, trans-acting factors, etc). Highlighting such phasic control, we propose a model in which *let-7-C* miRNAs collectively function as both a rheostat and as a switch but at distinct times (Fig 7). According to this model, *let-7* predominates during nervous system formation, where it shapes the temporal gradient of Chinmo. *let-7*-dependent attenuation of this dosage-sensitive transcription factor is responsible for the establishment of proper cell fate as neural progenitors divide. Subtle alterations in the rate of *let-7* accumulation may adjust the neuronal classes that comprise structures like the mushroom body, whose composition is known to be sensitive to environmental cues [48]. While *let-7* adjusts *chinmo*, *miR-125* in contrast switches *chinmo* off in post-mitotic neurons throughout the adult nervous system. This silencing of a juvenile neuronal marker maintains adult neuronal integrity, since forced Chinmo expression in adults leads to brain deterioration. While our model proposes that *miR-125* ensures complete silencing of *chinmo*, we cannot rule out the possibility that *miR-125* repression is alleviated under certain conditions in the adult (e.g. injury-induced repair) so that Chinmo can reprogram neurons to a juvenile state that is needed for certain adult functions. Thus, by independently regulating the same target during two different periods, *let-7* and *miR-125* miRNAs control cell fate establishment and maintenance, respectively.

Our model is based in part on results that *chinmo* repression is achieved predominantly by *miR-125* in adult brains, even though *let-7* is present. What accounts for the muted *let-7* activity that, while present, is not responsible for repression of a verified target? Perhaps *let-7* has many more targets than *miR-125* in the adult brain, since miRNAs with a larger repertoire of target genes have a weaker effect on each individual target [49, 50]. In addition, *let-7* targets may be highly expressed in the adult brain, thereby titrating away functional *let-7* and leading to its reduced effect on all its targets, including those that are co-targeted by both *let-7* and *miR-125*. Such a scenario is supported by increasing evidence that the effectiveness of a particular miRNA is influenced by the cellular concentration of available miRNA binding sites [51, 52]. Alternatively, perhaps *let-7* silencing requires cofactors that are only expressed during development. Future studies focused on the identification and characterization of the *miR-125-*independent targets of *let-7* should provide insight into the networks of *let-7* targets in adults.

### Significance and scenarios of miRNA co-targeting

While an overarching feature of miRNA regulation is that mRNAs are responsive to multiple miRNAs, our understanding of the biological significance of co-targeting is rudimentary. Supporting the apparently abundant co-targeting identified by miRNA binding site predictions [22–24, 28, 53], there are plenty of examples of multiple miRNAs that can when expressed one-by-one repress the same 3’UTR reporter. Such examples include repression of *mtpn* 3’UTR by any one of a trio of miRNAs (*miR-375*, *miR-125*, and *let-7b*) and repression of *cdkn1A/p21* 3’UTR by any one of a staggering 28 different miRNAs [53, 54]. Since the effects of combinations of miRNAs are rarely tested, such studies suggest that multiple miRNAs limit the spatial and/or temporal expression of targets and, in cells where they are co-expressed, may function redundantly with one another.

In addition to this simple scenario, there are hints of more complex combinatorial scenarios involving either cooperation or competition between co-targeting miRNAs. In the cooperative scenario, miRNAs with overlapping expression patterns lead to enhanced repression of co-targeted mRNAs in cells where they are expressed together. Examples of this include *miR-25* and *miR-221/222* co-repression of *p57* and *miR-148a* and the *miR-206* co-repression of *dmpk* [55, 56], although it is worth noting that the additive effects of these pairs of miRNAs is small though significant. Supporting such cooperative action, additional studies report that multiple binding sites, especially when they are within 15–35 bp of one another, lead to enhanced reporter repression [29, 57, 58]. Co-expressed miRNAs can also act competitively, as shown for *miR-184* and *miR-205* regulation of *ship2* [59]. In this scenario, *miR-184* does not have repressive activity itself but alleviates the repressive ability that *miR-205* exerts via an overlapping binding site, as shown by comparative analysis of *miR-205* alone versus in combination with *miR-184* as well as analysis of mutated 3’UTR reporters containing intact *miR-205* but mutant *miR-184* sites. In light of this *miR-184* function, systematic assays that have found that many miRNAs when expressed individually have no effect on a 3’UTR reporter do not rule out the possibility that these miRNAs function competitively with others [60, 61].

Phasic control expands the repertoire of known co-targeting functions, and emphasizes that co-targeting miRNAs may function at different times from one another and for different purposes. Phasic control may be particularly relevant to clustered miRNAs, since clustered miRNAs are enriched for co-targeting relationships [22, 28]. For example, the vertebrate *miR-17~92* cluster, like other miRNA clusters, targets multiple components of related networks and pathways of genes, including the TGF-β pathway [62, 63]. As with the *let-7-C* cluster in flies, members of polycistronic clusters, including the *miR-17~92* and *miR-1/miR-133* clusters, are differentially processed [64, 65]. The resulting differential accumulation of these co-targeting miRNAs, along with differential base pairing and turnover of co-transcribed miRNAs, may lead to the distinct temporal accumulations of processed miRNAs that are indicative of phasic control. Thus, the staggered accumulation of different miRNAs processed from the same polycistronic transcript over time may be an important feature controlling the progression of temporal features of cell and organismal biology.

### Conservation of co-targeting by *let-7-C* miRNAs

The biphasic regulation by *let-7-C* miRNAs may also be relevant to mRNAs co-targeted by *let-7-C* orthologues in other animals. These include *lin-28* and *lin-41*, which were originally identified in *C*. *elegans* as potential targets of both *let-7* and *lin-4*, the *C*. elegans *miR-125* orthologue [66, 67]. These co-targeting relationships are conserved to vertebrates, since mouse *lin-28* and *lin-41* 3’UTRs are responsive to altered levels of both *let-7* and *miR-125* [68–70]. The neurodevelopmental functions of these co-targetings has been extensively investigated and include, for example, *let-7*/*miR-125*-mediated repression of *lin-28* to control temporal identity during retinal neurogenesis in zebrafish [71, 72]. However, the careful dissection of the relative roles of *let-7* versus *miR-125* as well as their respective post-developmental functions awaits future investigation. While the *lin-28*/*let-7-C* relationship does not appear to be conserved to flies [73], the recent identification of a *let-7* and *miR-125* target with homology to *chinmo* suggests that regulation of a *chinmo* orthologue may be conserved [74]. This target, *hypermethylated in cancer 2 (hic2)*, encodes a BTB-zinc finger (BTB-ZF) transcription factor that contains multiple predicted *let-7* and *miR-125* sites in its 3’UTR. While reciprocal homology searching predicts equally good amino acid similarity between Chinmo and a number of mammalian BTB-ZFs including Hic2, the conservation of *let-7* and *miR-125* sites in the *hic2* 3’UTR but not other BTB-ZF 3’UTRs suggests that Hic2 is the mammalian orthologue of Chinmo. Thus, our results predict that mammalian *let-7* and *miR-125* regulate *hic2* in a biphasic manner.

### *let-7-C* miRNAs in aging and neurodegeneration

The persistence and gradual increase of *let-7-C* miRNAs during adult life may balance the various cellular demands needed for proper tissue and organismal homeostasis over time. Thus, the increasing levels of *let-7* that dampen stem cell function in aging tissue, found both in the mouse nervous system and the fly testis [35, 75], may be part of a general program that includes the neuronal maintenance function that this study explores. A conserved role for *let-7-C* miRNAs in such cell maintenance during adult life is supported by the requirement of *lin-4* for proper lifespan in *C*. *elegans* [2], since nematodes, like fly brains, exhibit limited cell proliferation during adulthood. In addition, *Drosophila let-7* and its target the *dp* transcription factor promote the maintenance of dopaminergic neurons in the adult brain by regulating the expression of pathogenic Leucine-Rich Repeat Kinase 2 [76]. Interestingly, changes in *miR-125b* have been linked to Alzheimer’s disease and vertebrate cerebellar neurodegeneration, although the molecular mechanisms underlying these changes have not been addressed [77, 78]. Taken together, this mounting evidence indicates that *let-7* and *miR-125* play critical neuroprotective roles in the aging brain so understanding their post-developmental functions in greater detail may be relevant to therapies for human neurodegenerative diseases, including Parkinson’s and Alzheimer’s diseases. In summary, our work has identified a novel *in vivo* mechanism by which multiple miRNAs repress a common target during distinct stages. Such differential regulation by subsets of co-expressed miRNAs should be considered for designing therapeutic strategies to treat diseases that are frequently caused by de-regulation of highly targeted mRNAs.

## Materials and Methods

### *Drosophila* husbandry

All flies were cultured on standard cornmeal medium at 25°C under 12 h light, 12 h dark cycles, except for flies analyzed in the temperature sensitive experiment presented in Fig 3. These flies were cultured in one of three regimens: at 18°C, at 29°C, and at 18°C until eclosion and then at 29°C thereafter. For steroid mediated UAS-transgene control using the Gene-Switch driver, flies were fed food containing 200μM RU-486 (Mifepristone, Cayman Chemicals, Ann Arbor MI). Staging of pupae and MARCM clone induction was performed as previously described [37, 38]. Unless otherwise noted, adult male flies of indicated ages were used for experiments.

### *Drosophila* genetics and *let-7-C* mutant strain construction

Detailed genotypes of all strains as well as the sources of the genetic mutations and transgenes used in the study are listed in S1 and S2 Tables, respectively. Transgenesis was performed by Rainbow Transgenic Services (Camarillo, CA) and BestGene, Inc. (Chino Hills, CA). The *let-7-C* mutant strains analyzed in this study (including *let-7-C*^*null*^, *let-7-C*^*hyp*^, *let-7-C*^*hyp*^ *rescue*, *ΔmiR-100*, *Δlet-7* and *ΔmiR-125* strains) were generated by crossing *w*^*1118*^*; let-7-C*^*GKI*^ */ CyO* strains to *w*^*1118*^*; let-7-C*^*KO2*^, *P{neoFRT}40A / CyO* strains in which one or both of these strains contained a rescuing transgene inserted on the third chromosome. Since this approach generated *trans*-heteroyzgous *let-7-C*^*GKI*^ */ let-7-C*^*KO2*^, *P{neoFRT}40A* animals, it eliminated the effect of any confounding recessive background mutations that might have accumulated on those chromosomes. In addition, because the differing rescuing transgenes were inserted at identical positions on the third chromosome, this approach ensured the pairwise comparison of strains that were otherwise as genetically similar to one another as possible. The detailed genetic scheme for generation of the transgenic samples is described in S1 Fig.

### Climbing and lifespan analyses

Climbing assays were performed as described previously [33]. Lifespan analysis was performed as previously reported [1, 3, 79] using *let-7-C* mutant flies that were generated as described above. Fifteen male flies (0–1 day old) were transferred to each vial. Flies were transferred to fresh food every 3 days at which time dead flies were counted and removed. The survival curves were plotted using Microsoft Excel. Statistical analysis was performed with the *Online Application for the Survival Analysis of lifespan assays* (OASIS) [80] and the p values were calculated using the log-rank (Mantel-cox) test. The number of flies used for each experiment are noted in the figure legends, and also included along with the median and maximum lifespans of the tested strains in S3 Table. S4 Table indicates the p values for curves shown in one or more panels. The numbers of flies used for each experiment have been noted in the figure legends. Experiments usually included two independent controls: *w*^*1118*^ as well as a *let-7-C* mutant strain containing a fully rescuing transgene. The *w*^*1118*^ survival curve was generated with flies that had been back crossed five times.

### Immunofluorescence

Immunofluorescence was performed as described previously [37, 38]. Primary antibodies included rat anti-Chinmo [38] (1:500), chicken anti-GFP (Rockland Immunochemicals, 1: 4000), rabbit anti-Woc [81] (gift from Maurizio Gatti 1:1000), rat anti-Elav (DSHB, 1:250) and mouse anti-Dachshund (DSHB, 1:100). For quantitating Chinmo levels, pixel intensity of 30 individual cells in single confocal sections of 5 independent dissected brains stained with anti-Chinmo and anti-Woc antibodies were quantified using ImageJ software. The expression of Chinmo was normalized to the pixel intensity of Woc and the average pixel intensity of one *Δlet-7-C* confocal section showing the highest pixel intensity was designated as 100 Arbitrary Units (AU). Samples whose staining was directly compared were prepared and imaged in parallel and under identical conditions.

For *c708a* neuron counts, mushroom bodies were optically sectioned in 0.5 μm increments, and the total number of neurons was determined by manually counting the number of GFP-positive cells section by section, ensuring that cells present on consecutive sections were counted only once. Statistical analysis was performed and histograms generated using GraphPad Prism software. P values were calculated using a two-tailed paired t test. Values are presented as mean ± SEM. All images were collected on a Leica SP5 confocal microscope (Light Microscopy Imaging Center, Indiana University, Bloomington IN). Confocal stacks were merged using Leica LSM software.

### Northern blot analysis and quantitative real time PCR

Total RNA was extracted with Trizol and treated with DNAse I. The reverse transcription was performed as described previously [82]. For analysis of miRNA copy number in Fig 4A, Kc-167 cells were incubated with 20E (5×10^−6^ M) at 25^0^ C for 24h before being washed with fresh medium. Reverse transcription (RT) was carried out on 25ng of total RNA using the Reverse Transcription miRNA Taqman assays (Applied Biosystem, Foster City, CA) specific for the miRNA (*dme-let-7* and *dme-miR-125*). Each cDNA sample was diluted 1:25 and real-time quantitative PCR (qPCR) was performed in duplicate using miRNA-specific primers/probe on a StepOnePlus Real Time PCR System (Applied Biosystem, Foster City, CA). For determination of copy number, we generated a standard curve for *let-7* and *miR-125* using a synthetic *let-7* and *miR-125* HPLC-purified RNA oligonucleotides synthesized by Integrated DNA Technologies (Coralville, IA) corresponding to the 22 nucleotide *miR-125-5p* (5’-rUrCrCrCrUrGrArGrArCrCrCrUrArArCrUrUrGrUrGrA-3’) and 21 nucleotide *let-7-5p* (5’- rUrGrArGrGrUrArGrUrArGrGrUrUrGrUrArUrArGrU-3’). For fold change analysis, individual values were normalized to 2S rRNA for Taqman miRNA assays and *kinesin* levels for Sybr green assays. For qRT-PCR analysis, oligos 2515, 2516, 2599, 2530, 2728, 2729 listed in S5 Table were used. Northern blot analysis was performed as described previously [37].

### Plasmid and transgenes

#### *Chinmo* transgenes

*pP{w+*, *UAS-chin*::*SV40}* contained the Chinmo open reading frame (ORF) flanked by *hsp70* 5’UTR and *SV40* 3’UTR and under the control of UAS sites. It was generated by PCR amplifying the Chinmo ORF with oligos 136 and 137 (see S5 Table for sequences) from reverse transcribed RNA generated from CNS tissue, then sequence verifying and subcloning the resulting fragment into the *BglII* and *NotI* restriction enzyme sites of plasmid *pUAST*. *pP{w+*, *UAS-chinmo*^*RNAi 148*^*}* encoded a short hairpin RNA (TGTGGGCTTTGAATACTACGC) targeting *chinmo*, designed based on rules described previously [83] and under the control of UAS sites. It was generated by subcloning the annealed oligos 1004 and 1005 (see S4 Table for sequences) into the *EcoRI* and *NheI* sites of plasmid *pWalium20* (TRiP at Harvard Medical School).

#### Sponge transgenes

Sponge constructs targeting *miR-100*, *let-7* and *miR-125* were designed based on Loya *et al*. [84] and Bejarano *et al*. [85]. A silencing cassette for each miRNA was synthesized by BioBasic, Inc that contained twenty miRNA complementary sequences separated by variable four-nucleotide linker sequences (see S6 Table for complete sequences). The entire cassette was subcloned into the *NotI* and *XbaI* sites of a modified *pValium10* plasmid (TRiP at Harvard Medical School, Boston, MA) and inserted into both attP40 and attP2 sites using phiC31 site-specific genomic integration. Resulting transformants were identified using *vermillion* as a transformation marker.

#### *let-7-C* locus transgenes

New transgenes were generated using previously reported P-element based transgenes containing either the full-length *let-7-C* locus or variants in which *miR-100*, *let-7*, and/or *miR-125* were specifically deleted [33]. These ~18kb fragments were excised using unique restriction sites *AvrII* and *XbaI* and subcloned into the *XbaI* site of a modified *pValium10* plasmid (TRiP at Harvard Medical School, Boston, MA)). Resulting plasmids were inserted into attP2 sites using phiC31 site-specific genomic integration and transformants were identified using *vermillion* as a transformation marker.

#### UAS-*let-7-C* constructs

These constructs were generated using the previously generated full length *pri-let-7-C* cDNA construct [82]. The hairpins corresponding to *pri-miR-100*, *pri-let-7* and *pri-miR-125* were deleted using splicing by overlap extension PCR. The oligo pairs 937/938, 939/940 and 941/942 were used to generate *ΔmiR-100*, *Δlet-7* and *ΔmiR-125* constructs, respectively. The *pri-let-7-C* cDNA was subcloned as an XhoI-KpnI fragment into pUAST-attB using the oligo pair 935/936. The oligo pair 1070/1071 was annealed to generate *pri-let-7 human let-7a2* and cloned into the XbaI site created after deleting *Drosophila melanogaster pri-let-*7. All PCRS were done with Pfu Polymerase.

#### Luciferase reporters

The nonmutated *chinmo* 3’UTR sensor has been described previously [38]. The constructs containing seed deletions of the four *miR-125* or six *let-7* sites were generated using the QuikChange Lightning Multi Site-Directed Mutagenesis Kit (Agilent Technologies, Santa Clara, CA) and oligonucleotides 978–981 and 972–977 respectively (see S5 Table for oligonucleotide sequences). The synthetic luciferase sensors were generated by annealing oligonucleotides with *NotI* and *XhoI* compatible ends (3046/3047, 3048/3049, 3050/3051 and 3052/3053). The annealed oligonucleotides (sequences in S5 Table) were cloned into a modified version of *pSiCheck2* (Promega Life Science, Madison WI). Fold repression was calculated relative to control samples transfected with empty vector instead of miRNA-encoding plasmids. The luciferase reporters used in Fig 2B were *psiCHECK* plasmids bearing six perfect sites for either *miR-100*, *let-7* or *miR-125* downstream of a *Renilla* luciferase gene were used and have been described previously [38].

#### Tagged protein plasmids

Plasmids encoding N-terminal Flag tagged version of Dicer was generated by recombining *pENTR-Dicer 1* (kind gift from Mikiko C. Siomi) with the *pAFW* gateway plasmid (T. Murphy; obtained from the Drosophila Genome Resource Center) using the LR Clonase enzyme (Life Technologies, Carlsbad CA), respectively. Flag tagged Drosha and Pasha constructs have been described previously [82].

### Histochemistry and scanning electron microscopy

For histochemistry, heads were fixed for 3h in AAF buffer (10% Formaldehyde, 5% Acetic Acid, and 85% Ethanol). The fixed tissue was serially passaged through 70% ethanol, 95% ethanol, 100% ethanol, and twice in Xylene for 45 minutes each. Following these incubations, the tissues were embedded in paraffin followed by sectioning. The 7 μm tissue sections were mounted on superfrost-plus slides (VWR International, Radnor PA) and processed for hematoxylin-eosin staining. For scanning electron microscopy, adult flies were serially passaged through 25% ethanol (10h), 50% ethanol (2h), 75% ethanol (2h), 100% ethanol (2h), 50% ethanol: 50% hexamethyldisilazane (HMDS)(3minutes) and 100% HMDS (3 minutes), coated with gold-palladium and viewed with a JEOL 5800LV SEM microscope.

### Luciferase reporter assays

*Drosophila* Kc-167 cells were cultured in CCM3 at 23°C. Cells were transfected in 48-well plates with 25 ng of *tub-Gal4* plasmid DNA, 25 ng *UAS-miRNA* plasmid DNA, and 25 ng of 3‘UTR-containing sensor plasmid DNA using Effectene (Qiagen). Luciferase assays were performed using the Dual-Luciferase reporter system (Promega Life Science, Madison WI). Transfections were performed in triplicates and resulting luciferase levels were averaged. Fold repression was calculated by dividing the ratio of *Renilla* luciferase and firefly luciferase in cells transfected with an empty pUAST attB plasmid with the ratio of *Renilla* luciferase and firefly luciferase in cells transfected with pUAST attB plasmid containing *let-7-C* cDNAs.

### MiRNA duplex transfection and determination of half-life of miRNAs

Si-miRNA duplexes were synthesized as single-stranded RNAs by Integrated DNA Technologies (Coralville, IA) with HPLC purification, and resuspended in duplex buffer (100mM potassium acetate, 30mM HEPES, pH 7.5) to a concentration of 100μM. Annealing was performed by incubating 50 μM complementary single-stranded RNAs at 92^**°**^C for 2 min and leaving them for 30 min at room temperature [86].

si miR-125 sense; 5’UCCCUGAGACCCUAACUUGUGAUU

si miR-125 antisense; 5’UCACAAGUUAGGGUCUCAGGGACU

si let-7 sense; 5’UGAGGUAGUAGGUUGUAUAGUCU

si let-7 antisense; 5’ACUAUACAACCUrArCrUrArCrCrUrCrArUrU

The miRNA duplexes were transfected using Dharmafect duo according to manufacturer’s instructions (GE Life Sciences, Lafayette CO). Briefly, 2.25 μl of mi-siRNA molecules (diluted to 4 μM in duplex buffer) was added such that the final concentration of each siRNA was 5 nM per well (the volumes indicated are for biological triplicate) in a 24 well plate. The cells were incubated at 25^0^ C for 5 h before being washed with fresh medium. Quantitative real time PCR was performed to measure the relative levels of the miRNAs in total RNA extracted from transfected cells at different time points. The half lives of *let-7* and *miR-125* were determined by exponential regression curve fitting using GraphPad Prism version 6 software.

### *In vitro* Drosha and Dicer processing assays

*Pri-let-7*, and *pri-miR-125* were generated by annealing oligos cloned into *pLitmus 28i*. DNA templates for transcription were generated by PCR with the T7 and 2162 oligo and were transcribed and labelled with ^32^UTP (Perkin Elmer, Waltham MA) using the T7 Megashortscript Kit (ThermoFisher, Cambridge MA). The transcript was purified by running the DNAse I treated reaction on a 4% denaturing PAGE gel and the gel piece corresponding to the labeled transcript was excised from the gel and eluted in a Eppendorf Thermomixer set at 400rpm and 37°C in a buffer containing 0.3M Sodium acetate, 0.2% Sodium dodecyl sulphate, and 1mM EDTA. The supernatant was precipitated in Ethanol. The precipitated RNA was refolded by heating at 95°C for 2 minutes followed by 37°C for 1 hour. A typical 25μL reaction contained 15μL of the Flag-Drosha-Pasha beads immunoprecipitate, 6.4mM MgCl_2_, 1 U/μL of Ribonuclease Inhibitor (ThermoFisher, Cambridge MA), and the refolded labeled transcripts (0.5 × 10^5^ cpm). The reaction mixture was incubated at 26°C for 30 to 90 min, and RNA was extracted by phenol followed by ethanol precipitation and analyzed on a 10% denaturing polyacrylamide gel.

*In vitro* dicing assays were typically carried out in 25μl lysis buffer, containing 5% (v/v) glycerol, 1 mM DTT, 0.1unit μl^−1^ RNasin Plus RNase Inhibitor (ThermoFisher, Cambridge MA), 1 nM 5′-radiolabeled substrate RNAs (GE Life Sciences, Lafayette CO; sequences listed below) and 25 nM Flag-tagged Dicer proteins. The reaction products were resolved by electrophoresis on 10% denaturing Page gel, detected by Typhoon phosphorimager and quantified by ImageQuant software (GE Life Sciences, Lafayette CO).

## Supporting Information

S1 Fig Scheme for Generation of Experimental Samples. This study compared flies that were generated using a scheme that ensured that they had similar genetic backgrounds. Flies that were analyzed (F14) were *trans*-heterozygous for two different *let-7-C* null alleles (indicated by red and yellow bars), ensuring that phenotypes were not due to recessive mutations on either *let-7-C* mutant chromosome. In addition, third chromosomes that contained differing rescuing transgenes (indicated by green bar) were derived in parallel from the same population of flies. Finally, all flies had a common X-chromosome (blue bar), derived from an isogenized stock. (**S1-1**) All rescuing transgenes, including the wildtype rescuing transgene as well as *let-7* and *miR-125* deleted versions, were injected into embryos from the same population of stock BL#25710 from the Bloomington Drosophila Stock Center. Resulting progeny were backcrossed twice to BL#32261 in order to select and balance *vermillion+* transformants (F1 and F2). Single transformants were subsequently backcrossed to an isogenized version of BL#3703 three times (F3-F5) in order to make balanced stocks with isogenized X chromosomes (F6). (**S1-2**) Stocks with differing rescuing transgenes were crossed to the same population of a stock that contained the *let-7-C*^*KO2*^ chromosome, an isogenized X chromosome, and two 3^rd^ chromosome balancers. The *let-7-C*^*KO2*^ stock used in F7 was generated in a similar fashion as the rescuing transgenes stocks, by backcrossing three times to an isogenized version of BL#3703. Resulting stocks (F8) had common X (blue), 2^nd^ (yellow) and 3^rd^ (green) chromosomes, and were used in F13 to generate the experimental strains. (**S1-3**) A second *let-7-C* allele, *let-7-C*^*GKI*^, was prepared by outcrossing twice to an isogenized stock, and then crossed to an isogenized stock containing a T(2:3) Cyo-TM6b compound chromosome. The *let-7-C* allele was selected based on mini-white, and the T(2:3) Cyo-TM6b balancer was selected based on the dominant Humoral marker. The resulting stock with a fixed second and third chromosome was amplified and used as the source for all virgins in the crosses that yielded the flies for analysis. (**S1-4**) Flies for analysis were generated by crossing virgins of the stock generated in F12 with males of stocks generated in F8 that harbored differing rescuing transgenes.
(TIF)

S2 Fig The vacuoles in aged *Δlet-7* and *ΔmiR-125* loss-of-function and Chinmo gain-of-function strains are predominantly localized to the central brain. (A) Depiction of major anatomical structures in a 3d aged *w*^*1118*^ brain. CB (central brain), Lo (lobula), LoP (lobula plate), Me (medulla), La (lamina) and Rt (retina). Scale bar: 20μm. (B-E) Quantitation of vacuoles in 40d aged brains of (B) *w*^*1118*^ (wt), *let-7-C*^*hyp*^, *let-7-C*^*hyp*^ *rescue* strains, (C) *let-7-C*^*null*^ *rescue*, *ΔmiR-100*, *Δlet-7*, and *ΔmiR-125* mutant strains, (D) *chinmo*^*1*^*; let-C*^*null*^ *rescue*, *chinmo*^*1*^*; ΔmiR-100*, *chinmo*^*1*^*; Δlet-7*, *chinmo*^*1*^*; ΔmiR-125*, *chinmo*^*RNAi*^*; ΔmiR-125* mutant strains, and (E) *elav*^*GS*^; *UAS-Chinmo* (-RU-486) and *elav*^*GS*^; *UAS-Chinmo* (+RU-486) strains.
(TIF)

S3 Fig Loss of *miR-125* and *let-7* enhance rCGG90 mediated retinal degeneration. (A-E) Scanning electron microscope (SEM) eye sections from 7d *GMR-Gal4* flies harboring a (A) *miR-125* sponge (*miR-125SP*), (B) a rCGG_90_ transgene (*rCGG*_*90*_), (C) a rCGG_90_ transgene along with a *miR-100* sponge (*miR-100 SP* + *rCGG*_*90*_), (D) a *let-7* sponge (*let-7SP* + *rCGG*_*90*_), or (E) a *miR-125* sponge (*miR-125 SP* + rCGG_90_).
(TIF)

S4 Fig *ΔmiR-125* mutants display late onset brain degeneration that is rescued by reducing *chinmo* levels. (A-L) Verification of *chinmo* knock down in *chinmo*^*RNAi*^ transgenic line. (A-F) Confocal images of 3d old adult brains immunostained for Chinmo (green) and Woc (red). The intensity of Chinmo immunostaining is reduced in brains harboring the *chinmo*^*RNAi*^ transgene (D-F) relative to the control (A-C). The genotype of the control in *A-C* is *let-7-C*^*GKI*^ */ let-7-C*^*KO2*^, *P{neoFRT}40A; {v+*, *let-7-C* ^*ΔmiR-125*^*}attP2 / +*, and the genotype displayed in *D-F* is *let-7-C*^*GKI*^ */ let-7-C*^*KO2*^, *P{neoFRT}40A; {v+*, *let-7-C* ^*ΔmiR-125*^*}attP2 / P{w+*, *UAS-chinmo*^*RNAi 148*^*}VK00033*. (G-L) *Elav-Gal4*, *UAS-mCD8*::*GFP* labeled wild type (G) and *UAS-chinmo*^*RNAi*^ (L) third instar larval clones generated in newly hatched larvae using the mosaic analysis with repressible cell marker (MARCM) technique and stained with Chinmo antibody. Absence of Chinmo staining in clones confirmed knockdown of *chinmo*. The genotype in G-I is *P{w+}elav[C155]*, *P{UAS-mCD8*::*GFP*.*L}LL4*, *P{hsFLP}1*, w[*]; P{tubP-GAL80}LL10 P{neoFRT}40A / P{neoFRT}40A; + and the genotype in J-L is *P{w+}elav[C155]*, *P{UAS-mCD8*::*GFP*.*L}LL4*, *P{hsFLP}1*, w[*]; P{tubP-GAL80}LL10 P{neoFRT}40A / P{neoFRT}40A; P{w+, *UAS-chinmo*^*RNAi 148*^*}VK00033 / +*. (M) Reducing dosage of *chinmo* decreases brain vacuolization in *ΔmiR-125* mutants. Histochemistry was performed on brain sections of 40d old *ΔmiR-125* mutants and *chin*^*1*^, *ΔmiR-125* mutants and the number of vacuoles were scored to assess brain morphology. Representative examples of brains sections are shown (vacuoles indicated by blue arrows) and the total vacuole number quantified from such sections of five independent brains is presented in Fig 2D.
(TIF)

S5 Fig *miR-125* is the predominant miRNA that silences *chinmo* in adult flies. (A-D) Confocal images of 3d old adult brains immunostained for Chinmo (green). No Chinmo expression was detected in brains of flies harboring either the wild type or the *ΔmiR-100* transgene (panels A and B). The level of Chinmo expression in *ΔmiR-125* mutants is much higher than in *Δlet-7* mutant adult flies (compare panels C and D). Genotypes used are the same as those listed for Fig 2B–2D in S1 Table.
(TIF)

S6 Fig Schematic of *let-7* and *miR-125* binding sites in the luciferase reporter. (A, B) Sequences and predicted base-pairing of *let-7* (green) and *miR-125* (red) binding sites in the luciferase sensors. Numbering is relative to the first nucleotide in the 3’UTR. Yellow boxes indicate the sequences that were deleted in the mutant constructs. The sites were designed so that the binding pattern was comparable between the miRNAs and the target sites. The nucleotides 1–9, 11–15, 19-21(*let-7*) and 19-22(*miR-125*) formed base-pairing interactions with the 3’UTR. The minimum free energy (mfe) calculated by RNAhybrid is indicated on the right of each binding site.
(TIF)

S7 Fig The decreased rate of processing of *miR-125* is compensated by its lower rate of decay. (A) Primary transcripts expressing wild type pri-*miR-125* does not undergo Drosha processing *in vitro*. *In vitro* processing of *pri-let-7* and *pri-miR-125* with purified Flag tagged Drosha-Pasha complex. The primary transcript (pri), 5’ flank (5’F), 3’ flank (3’F) and precursor (pre) are indicated on the right. Quantitation of the fraction processed is calculated as precursor/primary +5’F+3’F+precursor. (B) Western blot analysis of purified Flag tagged Drosha-Pasha(top panel) and Flag tagged Dicer 1(middle panel) used in Drosha and Dicer processing assays, respectively. (C) Expression analysis of Dicer 1 in UAS Dicer RNAi lines as determined by quantitative real time PCR of total RNA extracted from 10d old adult fly heads. Rp49 was used as a control for normalization. P-values determined by two-tailed paired t-test are denoted on top of the histogram. Assays were performed in triplicate for each experiment. Error bars, S.D. (D) MiRNA decay was calculated by quantitating the relative miRNA levels in Kc-167 cells transfected with miRNA duplexes and fitted exponential regression curve for *let-7* and *miR-125* indicates that the half life for *let-7* is lower than the half life of *miR-125*. The decay constant (λ) was extrapolated from the exponential decay curves of each biological replicate (n = 3 per time point), and the mean **±**S.D is shown. The half-life in hours (hr) was calculated by the formula ln2/λ.
(TIF)

S1 Table Genotypes used in this study. (DOCX)

S2 Table Sources of mutations and transgenes used in study. (DOCX)

S3 Table Median and maximum lifespan of strains used in the study. (DOCX)

S4 Table Comparison of survival curves by Log-Rank test. (DOCX)

S5 Table Primers used in this study. (DOCX)

S6 Table Sponge sequences used in this study. (DOCX)
