# Supplementary figures and images for: A let-7-to-miR-125 MicroRNA Switch Regulates Neuronal Integrity and Lifespan in Drosophila
Source: PLoS Genet. 2016 Aug 10;12(8):e1006247. doi: 10.1371/journal.pgen.1006247 (PMC4979967; doi:10.1371/journal.pgen.1006247)

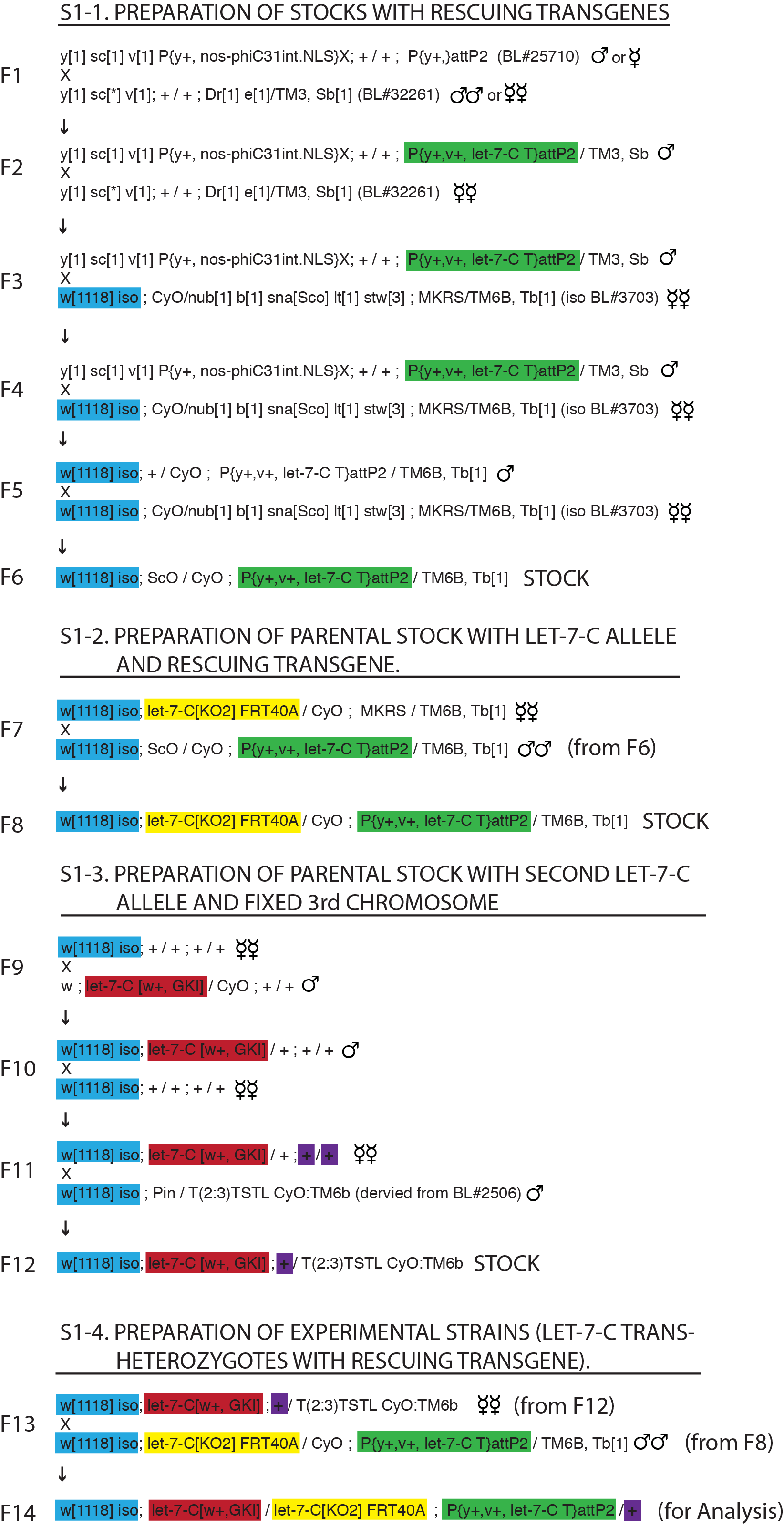

Supplement: S1 Fig — This study compared flies that were generated using a scheme that ensured that they had similar genetic backgrounds. Flies that were analyzed (F14) were trans-heterozygous for two different let-7-C null alleles (indicated by red and yellow bars), ensuring that phenotypes were not due to recessive mutations on either let-7-C mutant chromosome. In addition, third chromosomes that contained differing rescuing transgenes (indicated by green bar) were derived in parallel from the same population of flies. Finally, all flies had a common X-chromosome (blue bar), derived from an isogenized stock. (S1-1) All rescuing transgenes, including the wildtype rescuing transgene as well as let-7 and miR-125 deleted versions, were injected into embryos from the same population of stock BL#25710 from the Bloomington Drosophila Stock Center. Resulting progeny were backcrossed twice to BL#32261 in order to select and balance vermillion+ transformants (F1 and F2). Single transformants were subsequently backcrossed to an isogenized version of BL#3703 three times (F3-F5) in order to make balanced stocks with isogenized X chromosomes (F6). (S1-2) Stocks with differing rescuing transgenes were crossed to the same population of a stock that contained the let-7-CKO2 chromosome, an isogenized X chromosome, and two 3rd chromosome balancers. The let-7-CKO2 stock used in F7 was generated in a similar fashion as the rescuing transgenes stocks, by backcrossing three times to an isogenized version of BL#3703. Resulting stocks (F8) had common X (blue), 2nd (yellow) and 3rd (green) chromosomes, and were used in F13 to generate the experimental strains. (S1-3) A second let-7-C allele, let-7-CGKI, was prepared by outcrossing twice to an isogenized stock, and then crossed to an isogenized stock containing a T(2:3) Cyo-TM6b compound chromosome. The let-7-C allele was selected based on mini-white, and the T(2:3) Cyo-TM6b balancer was selected based on the dominant Humoral marker. The resulting stock with a [file pgen.1006247.s001.tif]

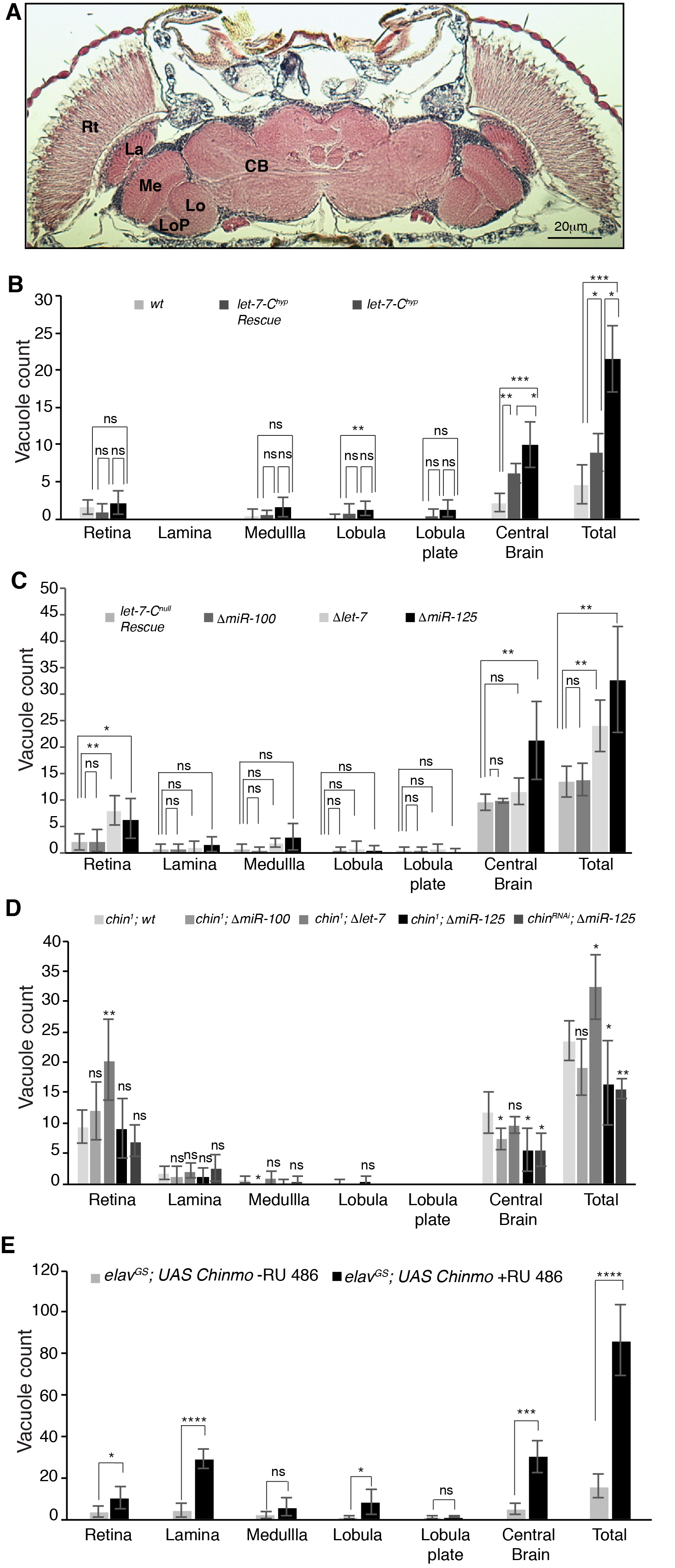

Supplement: S2 Fig — (A) Depiction of major anatomical structures in a 3d aged w1118 brain. CB (central brain), Lo (lobula), LoP (lobula plate), Me (medulla), La (lamina) and Rt (retina). Scale bar: 20μm. (B-E) Quantitation of vacuoles in 40d aged brains of (B) w1118 (wt), let-7-Chyp, let-7-Chyp rescue strains, (C) let-7-Cnull rescue, ΔmiR-100, Δlet-7, and ΔmiR-125 mutant strains, (D) chinmo1; let-Cnull rescue, chinmo1; ΔmiR-100, chinmo1; Δlet-7, chinmo1; ΔmiR-125, chinmoRNAi; ΔmiR-125 mutant strains, and (E) elavGS; UAS-Chinmo (-RU-486) and elavGS; UAS-Chinmo (+RU-486) strains. (TIF) [file pgen.1006247.s002.tif]

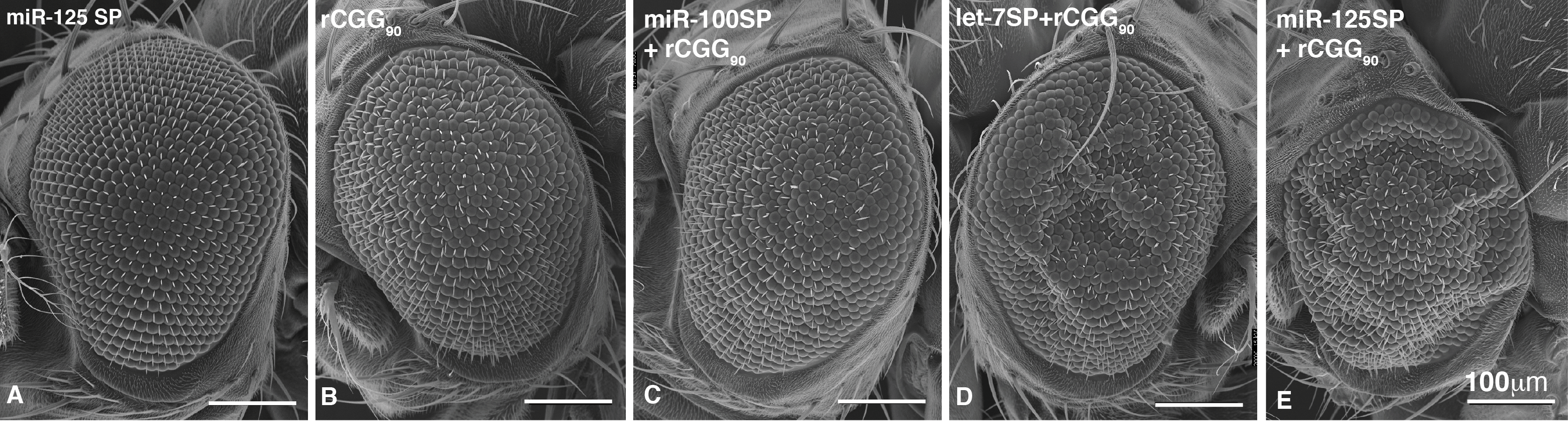

Supplement: S3 Fig — (A-E) Scanning electron microscope (SEM) eye sections from 7d GMR-Gal4 flies harboring a (A) miR-125 sponge (miR-125SP), (B) a rCGG90 transgene (rCGG90), (C) a rCGG90 transgene along with a miR-100 sponge (miR-100 SP + rCGG90), (D) a let-7 sponge (let-7SP + rCGG90), or (E) a miR-125 sponge (miR-125 SP + rCGG90). (TIF) [file pgen.1006247.s003.tif]

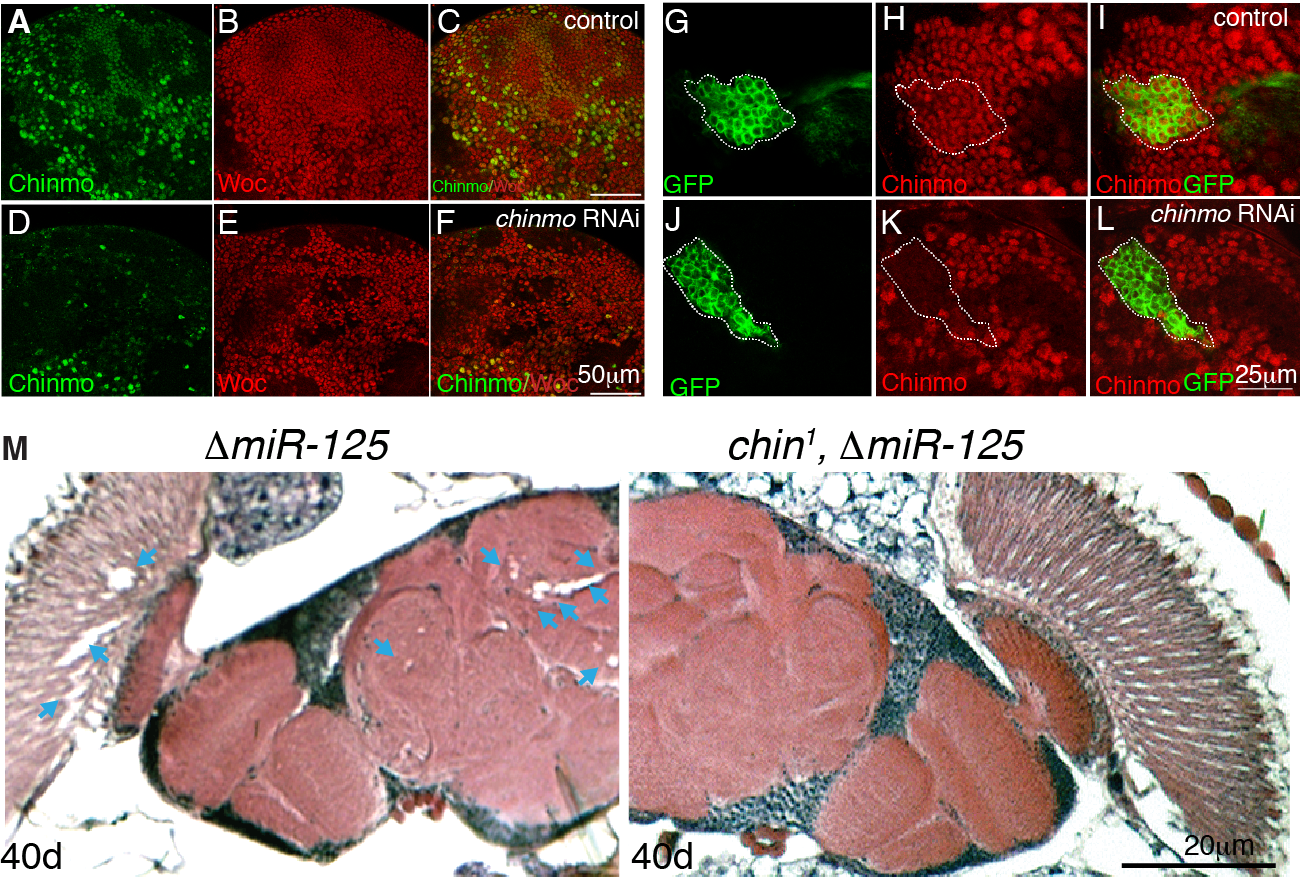

Supplement: S4 Fig — (A-L) Verification of chinmo knock down in chinmoRNAi transgenic line. (A-F) Confocal images of 3d old adult brains immunostained for Chinmo (green) and Woc (red). The intensity of Chinmo immunostaining is reduced in brains harboring the chinmoRNAi transgene (D-F) relative to the control (A-C). The genotype of the control in A-C is let-7-CGKI / let-7-CKO2, P{neoFRT}40A; {v+, let-7-C ΔmiR-125}attP2 / +, and the genotype displayed in D-F is let-7-CGKI / let-7-CKO2, P{neoFRT}40A; {v+, let-7-C ΔmiR-125}attP2 / P{w+, UAS-chinmoRNAi 148}VK00033. (G-L) Elav-Gal4, UAS-mCD8::GFP labeled wild type (G) and UAS-chinmoRNAi (L) third instar larval clones generated in newly hatched larvae using the mosaic analysis with repressible cell marker (MARCM) technique and stained with Chinmo antibody. Absence of Chinmo staining in clones confirmed knockdown of chinmo. The genotype in G-I is P{w+}elav[C155], P{UAS-mCD8::GFP.L}LL4, P{hsFLP}1, w[*]; P{tubP-GAL80}LL10 P{neoFRT}40A / P{neoFRT}40A; + and the genotype in J-L is P{w+}elav[C155], P{UAS-mCD8::GFP.L}LL4, P{hsFLP}1, w[*]; P{tubP-GAL80}LL10 P{neoFRT}40A / P{neoFRT}40A; P{w+, UAS-chinmoRNAi 148}VK00033 / +. (M) Reducing dosage of chinmo decreases brain vacuolization in ΔmiR-125 mutants. Histochemistry was performed on brain sections of 40d old ΔmiR-125 mutants and chin1, ΔmiR-125 mutants and the number of vacuoles were scored to assess brain morphology. Representative examples of brains sections are shown (vacuoles indicated by blue arrows) and the total vacuole number quantified from such sections of five independent brains is presented in Fig 2D. (TIF) [file pgen.1006247.s004.tif]

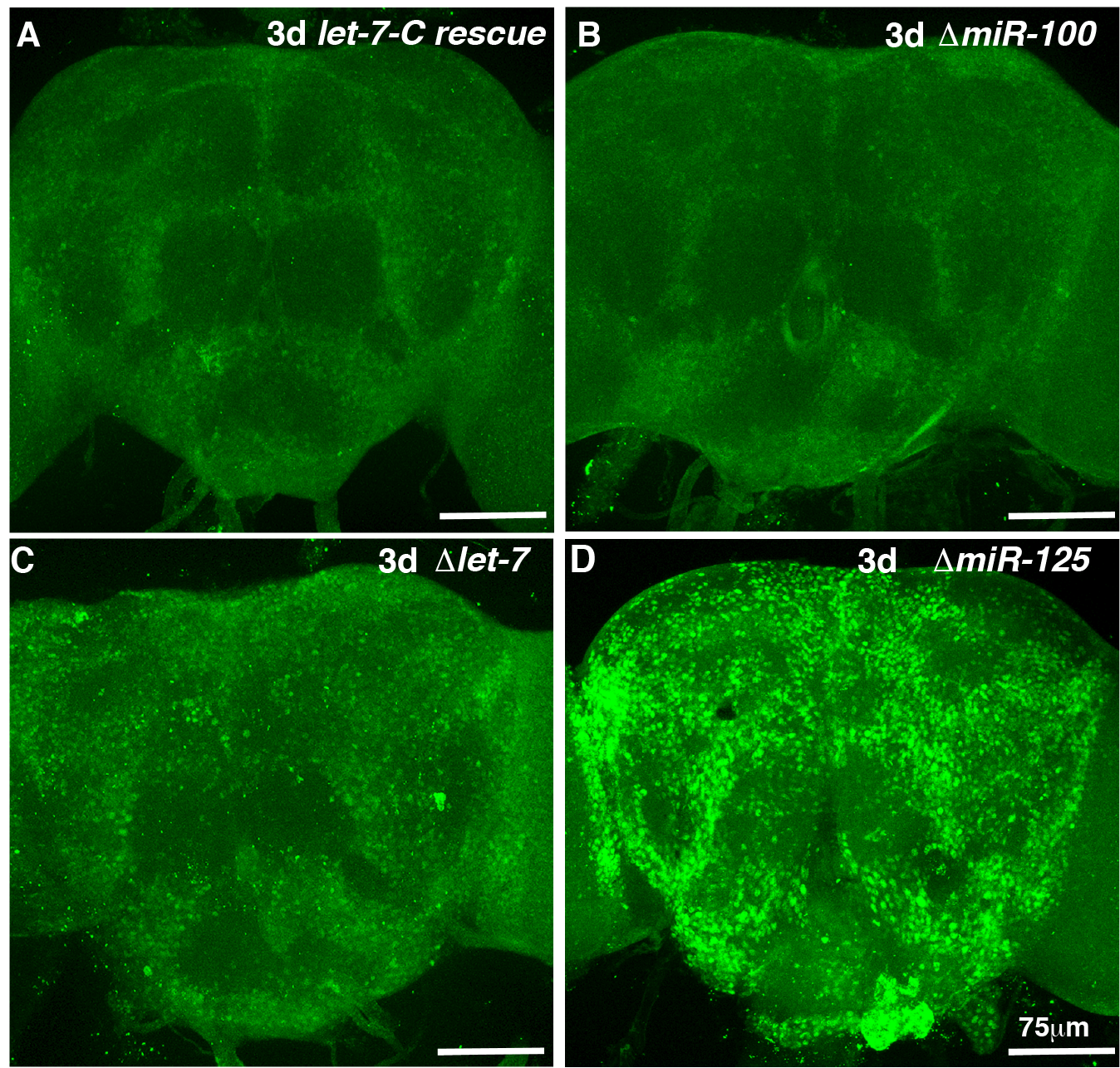

Supplement: S5 Fig — (A-D) Confocal images of 3d old adult brains immunostained for Chinmo (green). No Chinmo expression was detected in brains of flies harboring either the wild type or the ΔmiR-100 transgene (panels A and B). The level of Chinmo expression in ΔmiR-125 mutants is much higher than in Δlet-7 mutant adult flies (compare panels C and D). Genotypes used are the same as those listed for Fig 2B–2D in S1 Table. (TIF) [file pgen.1006247.s005.tif]

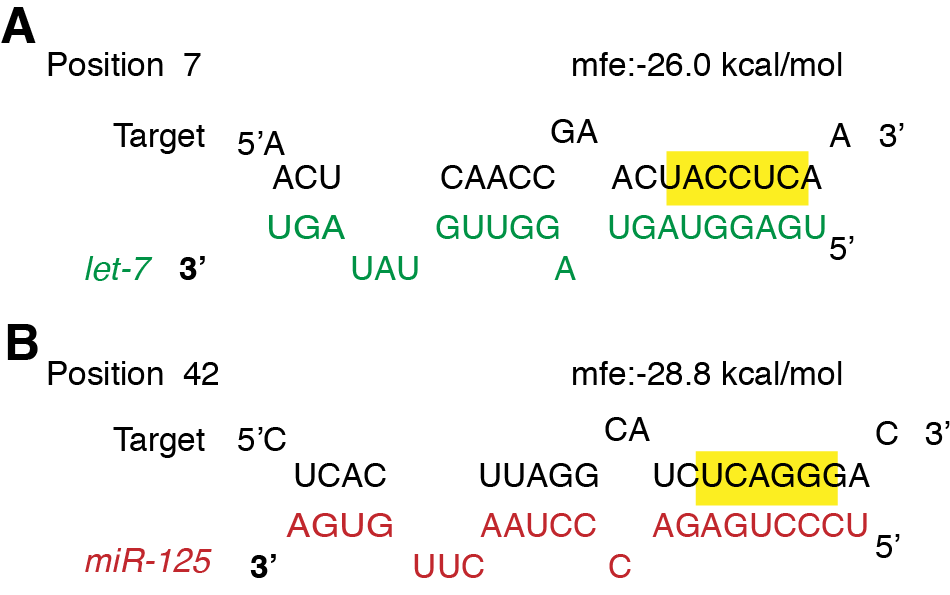

Supplement: S6 Fig — (A, B) Sequences and predicted base-pairing of let-7 (green) and miR-125 (red) binding sites in the luciferase sensors. Numbering is relative to the first nucleotide in the 3’UTR. Yellow boxes indicate the sequences that were deleted in the mutant constructs. The sites were designed so that the binding pattern was comparable between the miRNAs and the target sites. The nucleotides 1–9, 11–15, 19-21(let-7) and 19-22(miR-125) formed base-pairing interactions with the 3’UTR. The minimum free energy (mfe) calculated by RNAhybrid is indicated on the right of each binding site. (TIF) [file pgen.1006247.s006.tif]

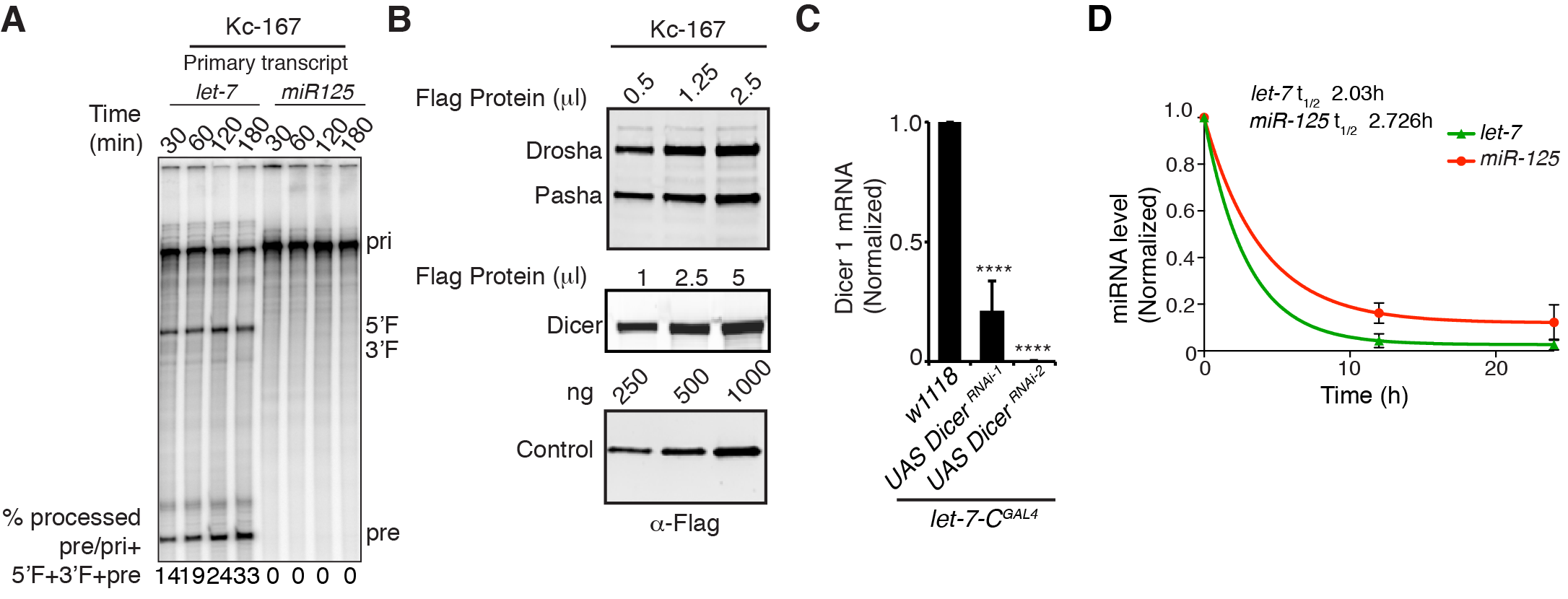

Supplement: S7 Fig — (A) Primary transcripts expressing wild type pri-miR-125 does not undergo Drosha processing in vitro. In vitro processing of pri-let-7 and pri-miR-125 with purified Flag tagged Drosha-Pasha complex. The primary transcript (pri), 5’ flank (5’F), 3’ flank (3’F) and precursor (pre) are indicated on the right. Quantitation of the fraction processed is calculated as precursor/primary +5’F+3’F+precursor. (B) Western blot analysis of purified Flag tagged Drosha-Pasha(top panel) and Flag tagged Dicer 1(middle panel) used in Drosha and Dicer processing assays, respectively. (C) Expression analysis of Dicer 1 in UAS Dicer RNAi lines as determined by quantitative real time PCR of total RNA extracted from 10d old adult fly heads. Rp49 was used as a control for normalization. P-values determined by two-tailed paired t-test are denoted on top of the histogram. Assays were performed in triplicate for each experiment. Error bars, S.D. (D) MiRNA decay was calculated by quantitating the relative miRNA levels in Kc-167 cells transfected with miRNA duplexes and fitted exponential regression curve for let-7 and miR-125 indicates that the half life for let-7 is lower than the half life of miR-125. The decay constant (λ) was extrapolated from the exponential decay curves of each biological replicate (n = 3 per time point), and the mean ±S.D is shown. The half-life in hours (hr) was calculated by the formula ln2/λ. (TIF) [file pgen.1006247.s007.tif]
