# Supplementary material for: A let-7-to-miR-125 MicroRNA Switch Regulates Neuronal Integrity and Lifespan in Drosophila
Source: PLoS Genet. 2016 Aug 10;12(8):e1006247. doi: 10.1371/journal.pgen.1006247 (PMC4979967; doi:10.1371/journal.pgen.1006247)
Supplement: S1 Table — (DOCX) [file pgen.1006247.s008.docx]

| **Fig.** | **Strain Name** | **Genotype** |
| --- | --- | --- |
| 1A-G | *Wt* | *w^1118^* |
|  | *let-7-C^null^* | *w^1118^; let-7-C^GKI^/ let-7-C^KO2^, P{neoFRT}40A* *; {v+, let-7-C ^Δlet-7-C miRNAs^}attP2 / +* |
|  | *let-7-C^hyp^* | *w^1118^; let-7-C^GKI^/ let-7-C^KO2^, P{neoFRT}40A* *; P{w+, let-7-Cp^3.3kb^::cDNA}VK00033 / {v+, let-7-C ^Δlet-7-C miRNAs^}attP2* |
|  | *let-7-C^hyp^ rescue* | *w^1118^; let-7-C^GKI^/ let-7-C^KO2^, P{neoFRT}40A* *; P{w+, let-7-Cp^3.3kb^::cDNA}VK00033 / P{v+, let-7-C}attP2* |
| 2C-F | *let-7-C^null^ rescue* | *w^1118^; let-7-C^GKI^ / let-7-C^KO2^, P{neoFRT}40A ; {v+, let-7-C}attP2 / +* |
|  | *ΔmiR-100* | *w^1118^; let-7-C^GKI^ / let-7-C^KO2^, P{neoFRT}40A* *; {v+, let-7-C ^ΔmiR-100^}attP2 / +* |
|  | *Δlet-7* | *w^1118^; let-7-C^GKI^ / let-7-C^KO2^, P{neoFRT}40A* *; {v+, let-7-C ^Δlet-7^}attP2 / +* |
|  | *ΔmiR-125* | *w^1118^; let-7-C^GKI^ / let-7-C^KO2^, P{neoFRT}40A* *; {v+, let-7-C ^ΔmiR-125^}attP2 / +* |
|  | *chin^1^ , wt* | *w^1118^; let-7-C^GKI^ / chinmo^1^, let-7-C^KO2^, P{neoFRT}40A* *; {v+, let-7-C}attP2 / +* |
|  | *chin^1^, ΔmiR-100* | *w^1118^; let-7-C^GKI^ / chinmo^1^, let-7-C^KO2^, P{neoFRT}40A* *; {v+, let-7-C ^ΔmiR-100^}attP2 / +* |
|  | *chin^1^, Δlet-7* | *w^1118^; let-7-C^GKI^ / chinmo^1^, let-7-C^KO2^, P{neoFRT}40A* *; {v+, let-7-C ^Δlet-7^}attP2 / +* |
|  | *chin^1^, ΔmiR-125* | *w^1118^; let-7-C^GKI^ / chinmo^1^, let-7-C^KO2^, P{neoFRT}40A* *; {v+, let-7-C ^ΔmiR-125^}attP2 / +* |
|  | *chin^RNAi^, ΔmiR-125* | *w^1118^; let-7-C^GKI^ / let-7-C^KO2^, P{neoFRT}40A* *; {v+, let-7-C ^ΔmiR-125^}attP2 / P{w+, UAS-chinmo^RNAi 148^}VK00033* |
| 3A-L | *+ rCGG_90_ , no sponge* | *w^1118/*^; P{w+, GMR-Gal4}, P{w+. UAS-rCGG_90_-EGFP} / + ; P{w+, tubP-Gal80[ts]}7 /+* |
|  | *+ rCGG_90_ , let-7SP* | *w^1118/*^; P{w+, GMR-Gal4}, P{w+, UAS-rCGG_90_-EGFP} / + ; P{w+, tubP-Gal80[ts]}7 / P{v+, UAS-let-7SP}attP2* |
|  | *+ rCGG_90_ , miR-125SP* | *w^1118/*^; P{w+, GMR-Gal4}, P{w+, UAS-rCGG_90_-EGFP} / + ; P{w+, tubP-Gal80[ts]}7 / ; P{v+, UAS-miR-125SP}attP2* |
|  | *- rCGG_90_ , no sponge* | *w^1118/*^; P{w+, GMR-Gal4} / + ; P{w+, tubP-Gal80[ts]}7 / +* |
|  | *- rCGG_90_ , let-7SP* | *w^1118/*^; P{w+, GMR-Gal4} / + ; P{w+, tubP-Gal80[ts]}7 / P{v+, UAS-let-7SP}attP2* |
|  | *- rCGG_90_ , miR-125SP* | *w^1118/*^; P{w+, GMR-Gal4} / + ; P{w+, tubP-Gal80[ts]}7 / ; P{v+, UAS-miR-125SP}attP2* |
| 4A-E | *elavGS*, *UAS-chinmo* | *P{elav-Switch.O}GS -1A / + ; P{elav-Switch.O}GS-3A, P{elav-Switch.O}GSG301 / P{w+, UAS-chin::SV40}* |
| 5A-J | *Wt* | *w^1118^* |
|  | *let-7-C wt* | *w^1118^; let-7-C^KO2^, P{neoFRT}40A* *; P{v+, let-7-C}attP2* |
|  | *Δlet-7* | *w^1118^; let-7-C^KO2^, P{neoFRT}40A* *; P{v+, let-7-C ^Δlet-7^}attP2* |
|  | *ΔmiR-125* | *w^1118^; let-7-C^KO2^, P{neoFRT}40A* *; P{v+, let-7-C ^ΔmiR-125^}attP2* |
|  | *Δlet-7-C* | *w^1118^; let-7-C^GKI^, P{neoFRT}40A* |
| 5K |  | *P{Gal4-c708a} , w^*^/ P{UAS-mCD8::GFP.L}LL4* |
|  | *let-7-C wt* | *P{Gal4-c708a} , w* / P{UAS-mCD8::GFP.L}LL4* ; *let-7-C^KO2^/ let-7-C^KO1^ ; P{v+, let-7-C }attP2* |
|  | *Δlet-7* | *P{Gal4-c708a} , w* / P{UAS-mCD8::GFP.L}LL4* ; *let-7-C^KO2^ / let-7-C^KO1^ ; P{v+, let-7-C ^Δlet-7^}attP2* |
|  | *ΔmiR-125* | *P{Gal4-c708a} , w* / P{UAS-mCD8::GFP.L}LL4* ; *let-7-C^KO2^/ let-7-C^KO1^ ; P{v+, let-7-C ^ΔmiR-125^ }attP2* |
|  | *Δlet-7*,  *ΔmiR-125* | *P{Gal4-c708a} , w* / P{UAS-mCD8::GFP.L}LL4* ; *let-7-C^KO2^/ let-7-C^KO1^ ; P{v+, let-7-C ^Δlet-7, miR-125^ }attP2* |
| 6A | Pupae and adults | *w^1118^* |
| 6D | *Dicer RNAi-1* | *w^1118^ ; {let-7-C^Δ3miR^::optGal4}attP40 / P{TRiP.HMS02594}attP40* |
|  | *Dicer RNAi-2* | *w^1118^ ; {let-7-C^Δ3miR^::optGal4}attp40 / + ; P{TRiP.HMS00141}attP2 / +* |
