# Supplementary material for: A let-7-to-miR-125 MicroRNA Switch Regulates Neuronal Integrity and Lifespan in Drosophila
Source: PLoS Genet. 2016 Aug 10;12(8):e1006247. doi: 10.1371/journal.pgen.1006247 (PMC4979967; doi:10.1371/journal.pgen.1006247)
Supplement: S2 Table — (DOCX) [file pgen.1006247.s009.docx]

| **Strain** | **Source** |
| --- | --- |
| *let-7-C^KO1^* | Ref [[25](#_ENREF_25)] |
| *let-7-C^GKI^* | Ref [[25](#_ENREF_25)] |
| *let-7-C^KO2^* | Ref [[30](#_ENREF_30)] |
| *chinmo^1^* | Ref [[35](#_ENREF_35)] |
| *P{w+, UAS-Chin::SV40}* | This study |
| *P{w+, UAS-chinmo^RNAi 148^}VK00033* | This study |
| *P{w+, UAS-miR-100SP}attP2* | This study |
| *P{w+ UAS-let-7SP}attP2* | This study |
| *P{w+, UAS-miR-125SP}attP2* | This study |
| *P{v+, let-7-C}attP2* | This study |
| *P{v+, let-7-C ^ΔmiR-100^}attP2* | This study |
| *P{v+, let-7-C ^Δlet-7^}attP2* | This study |
| *P{v+, let-7-C^ΔmiR-125^}attP2* | This study |
| *P{v+, let-7-C ^Δlet-7, miR-125^}attP2* | This study |
| *P{v+, let-7-C ^Δlet-7-C miRNAs^}attP2* | This study |
| *P{w+, let-7-Cp^3.3kb^::cDNA}VK00033* | Ref [[25](#_ENREF_25)] |
| *let-7-C^Δ3miR^::optGal4* | Ref [[30](#_ENREF_30)] |
| *P{GMR-Gal4}*, | kind gift from Peng Jin, Ref[[61](#_ENREF_61)] |
| *P{UAS-rCGG_90_-EGFP}* | kind gift from Peng Jin, Ref[[61](#_ENREF_61)] |
| *P{tubP-GAL80[ts]}7* | Bloomington Stock 7018 |
| *3XelavGS* | kind gift from Scott Pletcher |
| *P{w+, UAS-Dcr-1^RNAi-1^}attP40* | Bloomington Stock 42901 |
| *P{w+, UAS-Dcr-1^RNAi-2^}attP2* | Bloomington Stock 34826 |
| *P{C708A-Gal4},* | Bloomington Stock 50743 |
| *P{UAS-mCD8::GFP.L}LL4* | Bloomington Stock 5136 |
| *P{w+}elav[C155], P{UAS-mCD8::GFP.L}LL4, P{hsFLP}1, w[*] ; P{tubP-GAL80}LL10 P{neoFRT}40A* | Derived from Bloomington Stocks 5146 and 5192 |
