# Supplementary material for: A let-7-to-miR-125 MicroRNA Switch Regulates Neuronal Integrity and Lifespan in Drosophila
Source: PLoS Genet. 2016 Aug 10;12(8):e1006247. doi: 10.1371/journal.pgen.1006247 (PMC4979967; doi:10.1371/journal.pgen.1006247)
Supplement: S3 Table — (DOCX) [file pgen.1006247.s010.docx]

| Strain/Condition (number of flies) | Median lifespan (days) | Maximum life span (days) |
| --- | --- | --- |
| *w^1118^*(210) | 74 | 98 |
| *let-7-C^null^*(210) | 12 | 26 |
| *let-7-C^hyp^*(210) | 36 | 56 |
| *let-7-C^hyp^ rescue*(210) | 66 | 110 |
| *let-7-C^null^ rescue* (272) | 72 | 114 |
| *∆miR-100* (354) | 76 | 118 |
| *∆let-7* (202) | 36 | 62 |
| *∆miR-125* (337) | 38 | 68 |
| *chinmo^1^; Rescue*(172) | 58 | 118 |
| *chinmo1; ∆miR-100*(117) | 74 | 120 |
| *chinmo1; ∆let-7*(253) | 38 | 66 |
| *chinmo1; ∆miR-125*(153) | 52 | 100 |
| *chinmo RNAi; ΔmiR-125*(281) | 58 | 104 |
| *elavGS; UAS Chinmo*-RU-486 (162) | 66 | 94 |
| *elavGS; UAS Chinmo*+RU-486 (238) | 28 | 44 |
