# Supplementary material for: A let-7-to-miR-125 MicroRNA Switch Regulates Neuronal Integrity and Lifespan in Drosophila
Source: PLoS Genet. 2016 Aug 10;12(8):e1006247. doi: 10.1371/journal.pgen.1006247 (PMC4979967; doi:10.1371/journal.pgen.1006247)
Supplement: S4 Table — (DOCX) [file pgen.1006247.s011.docx]

| **Strains compared** | **P value** | **P value**  **summary** | **Are the curves significantly different (Yes/No)** | **Figure in manuscript** |
| --- | --- | --- | --- | --- |
| *w1118* and *let-7-C ^null^* | <0.0001 | **** | Yes | Fig. 1C |
| *w1118* and *let-7-C^hyp^* | <0.0001 | **** | Yes | Fig. 1C |
| *w1118* and *let-7-C^hyp^ rescue* | 0.5825 | ns | No | Fig. 1C |
| *let-7-C^hyp^*and *let-7-C^hyp^ rescue* | <0.0001 | **** | Yes | Fig. 1C |
| *let-7-C^null^ rescue* and *∆miR-100* | 0.4594 | ns | No | Fig. 2C |
| *let-7-C^null^ rescue* and *∆let-7* | <0.0001 | **** | Yes | Fig. 2C |
| *let-7-C^null^ rescue* and *∆miR-125* | <0.0001 | **** | Yes | Fig. 2C |
| *∆let-7 and ∆miR-125* | 0.7969 | ns | No | Fig. 2C |
| *let-7-C^null^ rescue* and *chinmo^1^; let-7-C^null^ rescue* | 0.0003 | *** | Yes | Fig. 2C and 2D |
| *∆miR-100 and chinmo^1^; ∆miR-100* | 0.6361 | ns | No | Fig. 2C and 2D |
| *∆let-7 and chinmo^1^; ∆let-7* | 0.0033 | ** | Yes | Fig. 2C and 2D |
| *∆miR-125 and chinmo^1^; ∆miR-125* | <0.0001 | **** | Yes | Fig. 2C and 2D |
| *∆miR-125 and chinmo^RNAi^; ∆miR-125* | <0.0001 | **** | Yes | Fig. 2C and 2D |
| *chinmo^1^;∆miR-125 and chinmo^RNAi^; ∆miR-125* | 0.0471 | * | Yes | Fig. 2D |
| *elav GAL4; UAS Chinmo –RU and elav GAL4 UAS Chinmo +RU* | <0.0001 | **** | Yes | Fig. 4B |
