# Supplementary material for: A let-7-to-miR-125 MicroRNA Switch Regulates Neuronal Integrity and Lifespan in Drosophila
Source: PLoS Genet. 2016 Aug 10;12(8):e1006247. doi: 10.1371/journal.pgen.1006247 (PMC4979967; doi:10.1371/journal.pgen.1006247)
Supplement: S5 Table — (DOCX) [file pgen.1006247.s012.docx]

| No. | Name | Sequence |
| --- | --- | --- |
| 136 | chinmo.4 f + bgl II | gcatagatctATGGATCCGCAGCAGCAGTTCTGCCTC |
| 137 | chinmo.5 r + not I | gcatgcggccgcCTATGGTGAATGATTGCTGGCTGCCGC |
| 948 | Nhe1 let-7 hairpin For | GATCtctagaaaaccacctagcaaaaaggactacaccaaggacctttttctctctggcaaattgaggtagtaggttgtatagtagtaattacacatcatactatacaatgtgctagctttctttgcttgactacaagccgcatttgat |
| 949 | Nhe1 let-7 hairpin Rev | CTAGCatcaaatgcggcttgtagtcaagcaaagaaagctagcacattgtatagtatgatgtgtaattactactatacaacctactacctcaatttgccagagagaaaaaggtccttggtgtagtcctttttgctaggtggtttG |
| 972 | 77.2 Δlet7bs.1 | cgatagcatgtgtgtatgcgtggagttgtataatgatagaacctag |
| 973 | 79.3 Δlet7bs.2 | ctgaatttcaattgtgaaactactgtatttccatgttttcgttagccagg |
| 974 | 78.3 Δlet7bs.3 | gccaaatgttgaatatgaaactatattaactgttgaaccacaagacaccac |
| 975 | 80.3 Δlet7bs.4 | ggcaaatatctgaaccaaaactattcgcatccaaacagacttaaccc |
| 976 | 76.8 Δlet7bs.5 | gagtctaatcaaactcaggaactcatataaggaaagaacctacctctttgtg |
| 977 | 79.3 Δlet7bs.6 | cgaactgcaatagtaaactgaactaaattttagccatagactgcgcagtg |
| 978 | 80.2 ΔmiR-125bs.1 | gttgagatgggttttatttgatccccaactgaatttcaattgtgaaac |
| 979 | 87.9 ΔmiR-125bs.2 | ttttaagtacgttggtggggagccttggattttggcgctgggtcagc |
| 980 | 75.9 ΔmiR-125bs.3 | acaatgtttaatacttagttgtcgttaattggagtaaccagcacacc |
| 981 | 81.7 ΔmiR-125bs.4 | gaatatattttacttttcggtttgcacaaacaaacagaggacgcgagcag |
| 1004 | CHUTRSHRNA.3T | ctagcagtccaactgaatttcaattgtgatagttatattcaagcatatcacaattgaaattcagttgggcg |
| 1005 | CHUTRSHRNA.3B | aattcgcccaactgaatttcaattgtgatatgcttgaatataactatcacaattgaaattcagttggactg |
| 968 | miR-125 Hairpin For | CTAGCaatattggcattggtgacatgtgcaaatgtttgtatggctgattccctgagaccctaacttgtgactttt  aataccagtttcacaagttttgatctccggtattggacgcaaacttgctgatgttagtaaaaaataaggcaaG |
| 969 | miR-125 Hairpin Rev | CTAGCttgccttattttttactaacatcagcaagtttgcgtccaataccggagatcaaaacttgtgaaactggtattaaaa  gtcacaagttagggtctcagggaatcagccatacaaacatttgcacatgtcaccaatgccaatattG |
| 1053 | Nhe1 let-7Hmir125B For | CTAGCaatattggcattggtgacatgtgcaaatgtttgtatggctgattgaggtagtaggttgtatagtagtaattacacatcatactatacaatgtgctagctttctggacgcaaacttgctgatgttagtaaaaaataaggcaaG |
| 1054 | Nhe1 let-7Hmir125B Rev | CTTGCCTTATTTTTTACTAACATCAGCAAGTTTGCGTCCAGAAAGCTAGCACATTGTATAGTATGATGTGTAATTACTACTATACAACCTACTACCTCAATCAGCCATACAAACATTTGCACATGTCACCAATGCCAATATTGCTAG |
| 2001 | Nhe I let-7 Hmir125LFor | CTAGCaaaccacctagcaaaaaggactacaccaaggacctttttctctctggcaaattgaggtagtaggttgtatagtacttttaataccagtttactatacaatgtgctagctttctttgcttgactacaagccgcatttgatG |
| 2002 | Nhe1 let-7Hmir125LRev | CATCAAATGCGGCTTGTAGTCAAGCAAAGAAAGCTAGCACATTGTATAGTAAACTGGTATTAAAAGTACTATACAACCTACTACCTCAATTTGCCAGAGAGAAAAAGGTCCTTGGTGTAGTCCTTTTTGCTAGGTGGTTTGCTAG |
| 2092 | Xba1 2001/3 PCR For | GATCtctagaaaaccacctagcaaaaaggactac |
| 2093 | Xba11 2001/3 PCR Rev | GATCTctagAATCAAATGCGGCTTGTAGTCAAGC |
| 2094 | Xba1 1053 PCR For | GATCTctagAaatattggcattggtgacatgtg |
| 2095 | Xba1 1054 PCR Rev | GATCtctagaTTGCCTTATTTTTTACTAACATCAG |
| 2162 | litmus Rev-2 xba | GCCTTGACTAGAGGGTACCAGAGCTCACCTAGG |
| 2500 | Rp49 QRT PCR For | CCCAAGGGTATCGACAACAGA |
| 2501 | Rp49 QRT PCR For | CGATGTTGGGCATCAGATACTG |
| 2515 | Pri miR-100 For | GGACCATTAACAGAAACCCGTAAA |
| 2516 | Pri MIr-100 Rev | CATAATGCCGGTCTTGTAACAGAT |
| 2728 | kin f 2 | GGCCAAGTCCATTGTTACGGA |
| 2729 | kin r 2 | CCTCCATCGTATGCGTTTTTCC |
| 2890 | Chinmo Long qRT PCR For | CGCAGTGACGAGATGAGCAT |
| 2891 | Chinmo Long qRT PCR Rev | AGAATAAAACGAAGTGGTAACGAAAGT |
| 2976 | Dicer1 QRT PCR For | AACCACGTCCGGGAACAAC |
| 2977 | Dicer1 QRT PCR Rev | TCGCAAAATTCGGATGCAAT |
| 3046 | Not1 let-7 Abrupt site 2 wt Age1-Sal1 miR-125 Chinmo site 2 wt For | ggccgcAACTCAACCGAACTACCTCAaccggtcacagtcgacTCACTTAGGCATCTCAGGGAc |
| 3047 | Xho1 let-7 Abrupt site 2 wt Age1-Sal1 miR-125 Chinmo site 2 wt Rev | tcgagTCCCTGAGATGCCTAAGTGAgtcgactgtgaccggtTGAGGTAGTTCGGTTGAGTTgc |
| 3048 | Not1 let-7 Abrupt site 2 mut Age1-Sal1 miR-125 Chinmo site 2 wt For | ggccgcAACTCAACCGAACAaccggtcacagtcgacTCACTTAGGCATCTCAGGGAc |
| 3049 | Xho1 let-7 Abrupt site 2 mut Age1-Sal1 miR-125 Chinmo site 2 wt Rev | tcgagTCCCTGAGATGCCTAAGTGAgtcgactgtgaccggtTGTTCGGTTGAGTTgc |
| 3050 | Not1 let-7 Abrupt site 2 wt Age1-Sal1 miR-125 Chinmo site 2 mut For | ggccgcAACTCAACCGAACTACCTCAaccggtcacagtcgacTCACTTAGGCATCAc |
| 3051 | Xho1 let-7 Abrupt site 2 wt Age1-Sal1 miR-125 Chinmo site 2 mut Rev | tcgagTGATGCCTAAGTGAgtcgactgtgaccggtTGAGGTAGTTCGGTTGAGTTgc |
| 3052 | Not1 let-7 Abrupt site 2 mut Age1-Sal1 miR-125 Chinmo site 2 mut For | ggccgcAACTCAACCGAACAaccggtcacagtcgacTCACTTAGGCATCAc |
| 3053 | Xho1 let-7 Abrupt site 2 mut Age1-Sal1 miR-125 Chinmo site 2 mut Rev | tcgagTGATGCCTAAGTGAgtcgactgtgaccggtTGTTCGGTTGAGTTgc |
| 1070 | pri Hs let-7-a2 For | CTAGCATGCTCCCAGGTTGAGGTAGTAGGTTGTATAGTTTAGAATTACATCAAGGGAGATAACTGTACAGCCTCCTAGCTTTCCTTGGGTCTTGCACTAG |
| 1071 | Pri Hs let-7-a2 Rev | CTAGTGCAAGACCCAAGGAAAGCTAGGAGGCTGTACAGTTATCTCCCTTGATGTAATTCTAAACTATACAACCTACTACCTCAACCTGGGAGCATGCTAG |
| 935 | Xho 1 let-7-C cDNA For | ccgctcgagttgcacacggcggttcgcgaatcgcg |
| 936 | Kpn1 let-7-C cDNA Rev | cggggtaccacttagaagtagcttgtagtttaaataca |
| 937 | del miR-100 Avr II For | gaccgccctaatgatttcttataCCTAGGtcgaaaaatcgacaaatggcggagtaagg |
| 938 | del miR-100 Avr II Rev | ccttactccgccatttgtcgatttttcgaCCTAGGtataagaaatcattagggcggtc |
| 939 | del let-7 Xba I For | gatatccagaagatcctttaaataccTCTAGAaaaagaatcccaatcgaactgcaccac |
| 940 | del let-7 Xba I Rev | gtggtgcagttcgattgggattcttttTCTAGAggtatttaaaggatcttctggatatc |
| 941 | del miR-125 Spe I For | gtatgtaaatgcaaccgggcatatgtaACTAGTaaacgaattccctttcaaggcac |
| 942 | del miR-125 Spe I Rev | gtgccttgaaagggaattcgtttACTAGTtacatatgcccggttgcatttacatac |
