# Supplementary material for: A let-7-to-miR-125 MicroRNA Switch Regulates Neuronal Integrity and Lifespan in Drosophila
Source: PLoS Genet. 2016 Aug 10;12(8):e1006247. doi: 10.1371/journal.pgen.1006247 (PMC4979967; doi:10.1371/journal.pgen.1006247)
Supplement: S6 Table — (DOCX) [file pgen.1006247.s013.docx]

| *miR-100SP (530bp)* | GCGGCCGCCACAAGTTCGctaTTACGGGTTCCCTCACAAGTTCGctaTTACGGGTTCCGCCACAAGTTCGctaTTACGGGTTCCGGCACAAGTTCGctaTTACGGGTTCCGACACAAGTTCGctaTTACGGGTTCGCCCACAAGTTCGctaTTACGGGTTCGGCCACAAGTTCGctaTTACGGGTTCGGGCACAAGTTCGctaTTACGGGTTCGGACACAAGTTCGctaTTACGGGTTCGACCACAAGTTCGctaTTACGGGTTCGGGCACAAGTTCGctaTTACGGGTTCCCTCACAAGTTCGctaTTACGGGTTCCGCCACAAGTTCGctaTTACGGGTTCCGGCACAAGTTCGctaTTACGGGTTCCGACACAAGTTCGctaTTACGGGTTCGCCCACAAGTTCGctaTTACGGGTTCGGCCACAAGTTCGctaTTACGGGTTCGGGCACAAGTTCGctaTTACGGGTTCGGACACAAGTTCGctaTTACGGGTTCGACCACAAGTTCGctaTTACGGGTTTCTAGA |
| --- | --- |
| *let-7SP (510bp)* | GCGGCCGCACTATACAACgatCTACCTCACCCGACTATACAACgatCTACCTCACCCTACTATACAACgatCTACCTCACCGGACTATACAACgatCTACCTCACGCGACTATACAACgatCTACCTCACGAGACTATACAACgatCTACCTCACAGTACTATACAACgatCTACCTCACTCGACTATACAACgatCTACCTCACTTGACTATACAACgatCTACCTCAGCGGACTATACAACgatCTACCTCACGAGACTATACAACgatCTACCTCACCCGACTATACAACgatCTACCTCACCCTACTATACAACgatCTACCTCACCGGACTATACAACgatCTACCTCACGCGACTATACAACgatCTACCTCACGAGACTATACAACgatCTACCTCACAGTACTATACAACgatCTACCTCACTCGACTATACAACgatCTACCTCACTTGACTATACAACgatCTACCTCAGCGGACTATACAACgatCTACCTCATCTAGA |
| *miR-125SP (530bp)* | GCGGCCGCTCACAAGTTAcatTCTCAGGGACCCGTCACAAGTTAcatTCTCAGGGACCCTTCACAAGTTAcatTCTCAGGGACCGGTCACAAGTTAcatTCTCAGGGACGCGTCACAAGTTAcatTCTCAGGGACGAGTCACAAGTTAcatTCTCAGGGACGATTCACAAGTTAcatTCTCAGGGACAGTTCACAAGTTAcatTCTCAGGGACTCGTCACAAGTTAcatTCTCAGGGACTTGTCACAAGTTAcatTCTCAGGGACGAGTCACAAGTTAcatTCTCAGGGACCCGTCACAAGTTAcatTCTCAGGGACCCTTCACAAGTTAcatTCTCAGGGACCGGTCACAAGTTAcatTCTCAGGGACGCGTCACAAGTTAcatTCTCAGGGACGAGTCACAAGTTAcatTCTCAGGGACGATTCACAAGTTAcatTCTCAGGGACAGTTCACAAGTTAcatTCTCAGGGACTCGTCACAAGTTAcatTCTCAGGGACTTGTCACAAGTTAcatTCTCAGGGATCTAGA |
